# Experimental Manipulation of Guided Attention to the Shoulder Movement Task in Clinical Dohsa-hou Induces Shifts in the Reactive Mode and Indicates Flexible Cognitive Control Performance

**DOI:** 10.3389/fpsyg.2022.785385

**Published:** 2022-04-06

**Authors:** Takuya Fujikawa, Russell Sarwar Kabir, Yutaka Haramaki

**Affiliations:** ^1^Graduate School of Education, Hiroshima University, Higashihiroshima, Japan; ^2^Graduate School of Humanities and Social Sciences, Hiroshima University, Higashihiroshima, Japan

**Keywords:** Dohsa-hou, cognitive control modes, self-regulation, dual mechanisms of control, AX-CPT, modified Stroop task

## Abstract

The empirical basis for self-control in Dohsa-hou as it relates to effects on cognitive processes has been explored in a few studies of the Japanese psychotherapy, but not under standardized conditions with a strong predictive theory of control. This study reports on a series of experiments with the Dual Mechanisms of Control framework to clarify the possible regulatory mechanism of Dohsa-hou by focusing on shoulder movement, a key body movement task used by practitioners across applied settings. Cognitive control was operationalized with the AX version of Continuous Performance Test (AX-CPT) paradigm for proactive control and a modified Stroop task paradigm for reactive control in a 3-arm parallel group trial study design. Healthy Japanese university students were assigned to a Dohsa-hou group that performed a shoulder movement task for few minutes, an active control group that performed a similar task, or a passive control group comprised of a resting condition. A total of 55 participants performed the AX-CPT and 57 participants performed the modified Stroop task before and after the group manipulation. In the AX-CPT, an increase in the error rate of AY (true cue-false probe) trial from pre- to post-test was observed in the passive control group only, and found to be marginally higher in the passive control group relative to Dohsa-hou group at post-test. This indicated that Dohsa-hou moderated the activation of proactive control by repeated AX-CPT performance. The error rate of the Proactive Behavioral Index did not differ from zero at post-test only in the Dohsa-hou group, indicating flexible cognitive control. In the modified Stroop task, there was no difference between congruent and incongruent trials at post-test for the Dohsa-hou group only, indicating the facilitation of reactive control. The evidence for a balancing effect for the Dohsa-hou-based shoulder movement task indicates that clients experience a form of continuous self-monitoring, which might reduce mind-wandering from their focus on movement execution combined with iterative verbal feedback from the therapist. Overall, the results of the present study suggest that the self-regulatory mechanism promoted in clinical Dohsa-hou emphasizes guided shifts in attention to the reactive mode toward a balance of cognitive control.

## Introduction

The Japanese psychotherapy known as Dohsa-hou (“body movement method”) has shown indicators of effectiveness in clinical settings and community applications. The growing evidence base suggests that the approach confers regulation over pain toward daily life functioning (Haramaki et al., 2019), relaxation-based coping with mood states as a form of stress management (Abe et al., 2019; Kuwashima and Yoshikawa, 2020), and body-mediated communication between clients and therapists (Fujino, 2017; Kawano and Fujino, 2021). Hypothesis formation has advanced about the role of cognition from experiments on the postural control of elderly adults (Adachi, 2015) and gains in interoceptive and proprioceptive awareness in Dohsa-hou applications toward understanding its fit with toolboxes of meditative movement (Kabir et al., 2019). However, at the behavioral performance level, precise relationships with delineations of its mechanism of change from true experimental designs are few, possibly due to research preferences in the development of Dohsa-hou that heavily featured a reliance on clinical case reports (Kamikura et al., 2020). In addition, while some studies have linked Dohsa-hou to known factors of body awareness (Fujino, 2012), indices of construct overlap with mindfulness (Konno, 2015), and mindful attention to the body (Kabir et al., 2019), the role of attention in Dohsa-hou has not been carefully studied under well-controlled, laboratory conditions. Moreover, observed improvements in inhibitory control in previous studies could not rule out mediating factors, such as the allocation of attentional resources tied to postural control that would constitute an indirect mechanism of change (Adachi, 2015; Konno, 2015). This study reports on a series of experiments with cognitive control modes to clarify the possible regulatory mechanism of Dohsa-hou by focusing on a key body movement task used by practitioners across applied settings of the approach. Cognitive control was experimentally evaluated with the AX version of the Continuous Performance test (AX-CPT) paradigm for proactive control and a modified Stroop task paradigm for reactive control, explained according to Dual Mechanisms of Control (DMC) theory (Braver, 2012).

### Background and Theoretical Advances From Research on Dohsa-hou

Studies on relationships between physical movement and psychological functioning illustrate a common basis of self-regulatory change and point to a role for the toolbox of *meditative movement*, a broad term for movement-based and embodied contemplative practices for mental and physical health (Payne and Crane-Godreau, 2013; Schmalzl et al., 2014; Schmalzl and Kerr, 2016). Under this umbrella is Dohsa-hou, stipulated by Gosaku Naruse in Japan, which is an experiential therapy that uses body movements for clients to achieve degrees of self-regulation (Naruse, 1997; for review, see Chervenkova, 2017; Kabir et al., 2018; Kamikura and Shimizu, 2021). Dohsa-hou has been reportedly useful for enhancing the mind and body connection for athletes (Hoshino, 1997) and differently abled sportsmen (Dadkhah, 1998). It is important to note, however, that Dohsa-hou is distinct from and should not be conflated with forms of exercise therapy (Kamikura and Shimizu, 2021). The fit of Dohsa-hou among mainstream body psychotherapies is debated, although a coherent overlap has been identified with the main themes of integrated body psychotherapy (Kamikura et al., 2020). These include a sense of holism from a body–mind connection, being agentic and able, being unique, worthy, and accepted according to one’s self-view, changing interactions and engaging in interpersonal contact, being a part of a group and feeling integrated, and having hope and investment in the future (Galbusera et al., 2019; Kamikura et al., 2020). The method shares characteristics of other proprioceptive approaches that provide perceptual goals to integrate intentional motor behavior with movement-related feedback for client comparison (Tous-Ral et al., 2012).

Within Japan, Dohsa-hou has developed with many of these characteristics in mind, especially in applications that were implemented for depression (Koga, 2001), anxiety (Ikeda, 2001), schizophrenia (Tsuru, 1993; Kamikura et al., 2020), interpersonal engagement in the form of joint attention to task performance and other-related recognition (Kawano and Fujino, 2021), and decreases in maladaptive behaviors and internalizing symptoms among individuals with developmental conditions (Fujino, 2017). Outside of Japan, the main themes have also appeared in comparisons between Dohsa-hou and the effectiveness of a similarly described movement-mediated approach to body awareness in the form of the Alexander Technique (Pour Kamali et al., 2018). In a study by Pour Kamali et al. (2018), this comparison was successfully conducted in a clinical population with outcomes related to mental health and social adjustment in terms of interpersonal functioning. The study observed effectiveness in favor of Dohsa-hou for improving levels of subjective happiness and hope with university students (Pour Kamali et al., 2018). Across findings in clinical and community settings, Dohsa-hou especially leverages the experience of motor resonance as a form of self-control and the use of the movement tasks for deliberately enacted physical relaxation as behavioral activation, which contributes to the regulation of daily life functioning (Haramaki et al., 2019), and the management of stress responses (Abe et al., 2019; Kuwashima and Yoshikawa, 2020).

While the practice has advanced for decades after work by influential scholars, many points about Dohsa-hou as a body psychotherapy are still relatively unknown to global scholarship and less integrated into formal training for psychotherapists even within Japan (Kamikura and Shimizu, 2021). Dohsa-hou researchers do not yet agree on its core theory of embodiment, although some confirmatory studies based on Naruse’s theory, which was derived from clinical observations, have been conducted (Takeuchi, 2017). For other theories, body consciousness based on private and public forms of self-consciousness (Dadkhah, 1998), a broad form of multidimensional body awareness (Fujino, 2012), an assessment-based focus on Mehling et al., interoceptive attention tendencies (Kabir, 2019), and an integrated view based on Gallagher’s conception of the self (Kamikura, 2017), have been proposed and explored. The level of conscious awareness (e.g., as reflexive or volitionally attending) raised by clients in a clinical setting remains an open question for the role of attention in Dohsa-hou (Kabir et al., 2018; Kamikura and Shimizu, 2021).

Nevertheless, a consensus around a broad form of body awareness continues to be theorized *via* the clinical observation process for Dohsa-hou as a means to pay conscious attention to bodily processes from exposure to and acquisition of the skills in the psychotherapy, such that the client: (1) forms a mental image of an intended movement or movements, (2) effortfully enacts them with actions of the limbs, (3) notices a change in feeling states from the feedback and evaluation of the performance of the movement, and (4) adjusts the body movements in an iterative process. This process pares down the suite of movement tasks into simple instrumental actions that can be performed by clients and observed by therapists. Crucially, the tasks scaffold into increasingly more expansive sections of the body and increasing levels of body awareness, and the experience of relaxation or body control reciprocally leads to trust in the therapeutic alliance, as verbal instructions and feedback are concurrent with the repertoire of movement tasks in Dohsa-hou clinical practice (Takeuchi and Takemoto, 2017). Fujino (2012) underscored the role of feedback from the therapist during the performance of movement tasks in Dohsa-hou as the first step in the positive psychological process, and the movement tasks themselves have shown flexible application into brief session interventions (Abe et al., 2019; Kuwashima and Yoshikawa, 2020).

### Empirical Research on Dohsa-hou and Its Configurations

While there are several instances of self-reported changes in psychological variables in studies of Dohsa-hou, there are fewer reports of basic research findings from experimental studies, and most relate to the relaxation-related benefits of the psychotherapy. For example, Konno (1999) reported effects of movement task-based relaxation in Dohsa-hou on visual acuity and auditory responses from experimental control of *tokeai dohsa* (“feeling of warmth or melting in relaxation from the movement”). In a subset configuration of Dohsa-hou known as Self-Active Relaxation Therapy (Ohno, 2005; Kabir et al., 2018) which uses the same movement tasks as Dohsa-hou, a basic finding with electromyography (EMG) was performed by Mukasa and Ohno (2016), who observed significant decreases in EMG activity in terms of mean frequency and root-mean square values of the EMG signal after performing the Dohsa-hou shoulder relaxation task. Finally, Kuwashima et al. (2021) used heart-rate variability analysis and observed that Dohsa-hou relaxation in the supine position enhanced cardiac parasympathetic activity. Dohsa-hou research has been strongly tied to findings of clinical significance, especially regarding rehabilitation processes for individuals with cerebral palsy (Dadkhah, 1998; Imura, 2016). More empirical research, however, is needed to match Naruse’s original and founding formulation of the role of “self-control” in the psychotherapeutic process.

Outside of the factor analytic study by Takeuchi (2017), less is understood about the fundamental role of self-control in Dohsa-hou in non-clinical settings, and there is a crossover need to understand the effects of cognitive processes with the movement tasks used throughout approaches. There are only two empirical studies investigating the role of self-control in Dohsa-hou in terms of a cognitive model. The first is a study by Adachi (2015) who operationalized Stroop task performance as an indicator of inhibition, who found it in favor of postural control benefits from Dohsa-hou related to balance among the elderly. The study also reported a reduction in Stroop processing times for the Dohsa-hou group condition and state anxiety decreases measured *via* STAI-S. Similarly, the second study by Konno (2015) also observed reduced state anxiety and Stroop interference in a group exposed to Dohsa-hou relaxation, especially for the incongruent condition. These two studies laid the groundwork for basic research on Dohsa-hou and cognitive processes.

Both studies focused only on the effects of reactive inhibition in their choice and manipulations of Stroop test. However, proactive contributions to inhibition would also be expected to play a role, especially among adult populations, to which a proactive dominance is developed (Munakata et al., 2012; Gonthier et al., 2019). In addition, their choice of Stroop tests captured simultaneous processing, which might have less advantages than task design choices that feature sequential processing, as the former lacks the opportunity to securely disambiguate proactive processing based on contextual information (Lesh et al., 2013). Finally, Adachi (2015) relied on a relatively traditional measurement strategy for the Stroop task by using a low-resolution, paper-based stimulus presentation, and a stopwatch to capture response times. Neither study performed rigorous tests of reliability for the Stroop experimental paradigm, and the lack of reporting and standardization makes it difficult to compare the results to other studies. The primary research aim of our study is to address these issues in Dohsa-hou research with a strong theoretical framework, the Dual Mechanisms of Control, and the well-validated and standardized experimental paradigms of the AX-CPT and modified Stroop task associated with investigations of cognitive control modes.

### Strengths of the Dual Mechanisms of Control Framework and AX-CPT Paradigm

In contrast to the theoretical pluralism and experimental limitations of Stroop tasks used in previous Dohsa-hou studies, the DMC framework distinguishes between goal-directed and stimulus-driven processing and allows researchers to make predictions about capability-based individual differences in cognitive processes and attention. Braver (2012) made crucial distinctions about cognitive control by operationalizing its variability and proposed two summaries for the modes. The first is the *early selection* of goal-relevant information that relies upon anticipating and preventing interference before it occurs or proactive control. The second is the *late correction* mode involved in detecting and resolving interference after it occurs. The AX-CPT is an adapted version of the Continuous Performance Test which was designed to induce cognitive control by increasing demands from contextual cues (Gonthier et al., 2016). Gonthier et al. (2016) provided experimental evidence for the AX-CPT paradigm to induce shifts in the proactive mode, which gave support for the DMC framework, especially related to the effects of strategy training. Cooper et al. (2017) further evaluated empirical datasets using the AX-CPT with detailed psychometric analyses, indicating important characteristics for study design. Furthermore, Yang et al. (2018) extended the predictive validity of the paradigm by manipulating state anxiety and the performance of the AX-CPT. These studies and others have shown the strengths of the approach to evaluating cognitive control for context processing and goal maintenance.

The DMC and AX-CPT are advantageous for the empirical study of Dohsa-hou for several reasons. These include the fact that studies of mindfulness, which is considered a reference point for Dohsa-hou constructs in previous studies (Konno, 2015; Kabir et al., 2018), have incorporated the modes of cognitive control with the AX-CPT (Li et al., 2018). For example, Chang et al. (2018) applied the AX-CPT and observed a “balancing” effect of flexibility in using the two control modes for more mindful individuals and those in a brief mindfulness manipulation group relative to controls. This suggested that mindfulness enhances proactive and reactive control and leads to flexible performance on the task. Another reason is that the AX-CPT has been investigated in the context of the anticipating movement. Liebrand et al. (2017) studied the relationship between movement and cognitive control in terms of proactive and reactive motor inhibition and showed that sensorimotor activity measured by EEG was predicted by the DMC framework. These studies indicate the potential for the DMC and AX-CPT, in addition to the Stroop task, to elucidate a possible role for Dohsa-hou movement tasks and the characteristics of their concomitant psychological process of attention. This conforms to our key research question and study purpose.

### Purpose of the Study

Given this review, the self-regulatory mechanism of Dohsa-hou involves an experientially generated source of internal feedback that is attenuated by external input from therapist-driven verbal instructions. The reinforcement of this use of guided attention to body movement execution might be expected to reduce mind-wandering due to the focus on task performance provided as feedback by the therapist. The feedback loop for the “harmonious” relationship between movement performance and psychological attention to the body would also be expected to recruit the flexible application of cognitive control.

Thus, this study aims to test the empirical basis for the role of attention in self-regulation from exposure to a movement task with verbal feedback in Dohsa-hou with a three-arm parallel group trial study design with two well-validated paradigms from the task battery of cognitive control (AX-CPT and modified Stroop task) and randomized participant assignment under well-controlled laboratory conditions. In a novel and more severe test for Dohsa-hou research, a resting condition is used as a passive control group, and an exercise movement task is used as an active control group for comparison with the shoulder movement task as the treatment group. Statistical analysis was planned for comparisons with group, time, and trial type as factors.

We hypothesized that under the conditions of the Dohsa-hou movement task with guided feedback, participants would demonstrate flexible cognitive control performance. Based on the DMC framework, we proposed the following three hypotheses:

*H1*: The proactive control mode as measured by the AX-CPT will not differ between the Dohsa movement task condition group and the control movement task condition groups.

*H2*: The Proactive Behavioral Index (PBI) for response times and error rates will indicate a balance between modes of cognitive control performance in the Dohsa task condition group relative to the other conditions.

*H3*: The reactive control mode as measured by the modified Stroop task will be increased for the Dohsa task condition group relative to the control movement task groups.

## Materials and Methods

### Participants

This study was conducted after receiving approval from the ethical review committee of the Graduate School of Humanities and Social Sciences, Hiroshima University. The participants were healthy Japanese university students, as screening questions indicated that none had been diagnosed with or were currently undergoing treatment for physical or mental illnesses. They also did not indicate excessive sleep deprivation, alcohol consumption, or smoking habits in the screening.

Participants were randomly assigned to the Dohsa-hou group and the active control group. To prevent arbitrary assignment by the experimenter, the order of group assignment was pre-determined. The passive control group was additionally recruited as an added arm after data from the Dohsa-hou and active control groups had been obtained to account for the possible effect of habituation due to repeated measures of cognitive tasks. Participants were blinded to group assignment. The experimenter facilitated the movement task conditions and was therefore not blind to the order of task assignment.

Because the two cognitive tasks were measured on separate days, participants had to participate in the experiment twice. Some participants participated only on the first day of the experiment, resulting in differences in the number of participants who performed each task. A total of 55 participants performed the AX-CPT. Among this sample, four participants (two in the Dohsa-hou group and two in the passive control group) who had an error rate of 50% or more on any trial in either the pre- test or post-test were excluded from the analysis. The final sample consisted of 12 participants (seven males, *M*_age_ = 23.8, SD = 1.8) in the Dohsa-hou group, 13 participants (eight males, *M*_age_ = 24.2, SD = 1.9) in the active control group, and 26 participants (10 males, *M*_age_ = 22.7, SD = 1.5) in the passive control group.

A total of 57 participants performed the modified Stroop task. Among this sample, four participants (two in the Dohsa-hou group, one in the active control group, and one in the passive control group) who had an error rate of 50% or more on any trial in either the pre-test or post-test were excluded from the analysis. The final sample consisted of 13 participants (six males, *M*_age_ = 23.7, SD = 1.8) in the Dohsa-hou group, 12 participants (seven males, *M*_age_ = 24.3, SD = 2.0) in the active control group, and 28 participants (12 males, *M*_age_ = 22.6, SD = 1.5) in the passive control group.

### Cognitive Tasks

The AX-CPT and modified Stroop task were performed using PsychoPy (Version 3.2.1; Peirce et al., 2019) running on a 15.6-inch laptop computer (Fujitsu, Lifebook AH77/J). The following sections detail the task processing steps in the study design and implementation.

#### AX-CPT Design and Procedures

We followed the AX-CPT procedure developed by Braver (2012) to perform the experiment. AX-CPT is a task that presents a letter as a cue stimulus and is held in order to produce an appropriate response with a probe stimulus. The AX-CPT consisted of four trial types: AX, AY, BX, and BY, with AX trials as the target trial type. Since the proportion of AX trials is higher than that of the other trial types, the expectation that the cue stimulus “A” would be followed by the probe stimulus “X” is induced. The AY trial thus requires inhibition of the dominant response, presenting a lot of errors and long response times (RTs). For BX trials, processing is optimized by retaining the cue stimulus, resulting in fewer errors and shorter RTs. These features indicate a tendency to perform preparatory processing according to contextual information (i.e., proactive control).

**Figure 1:**
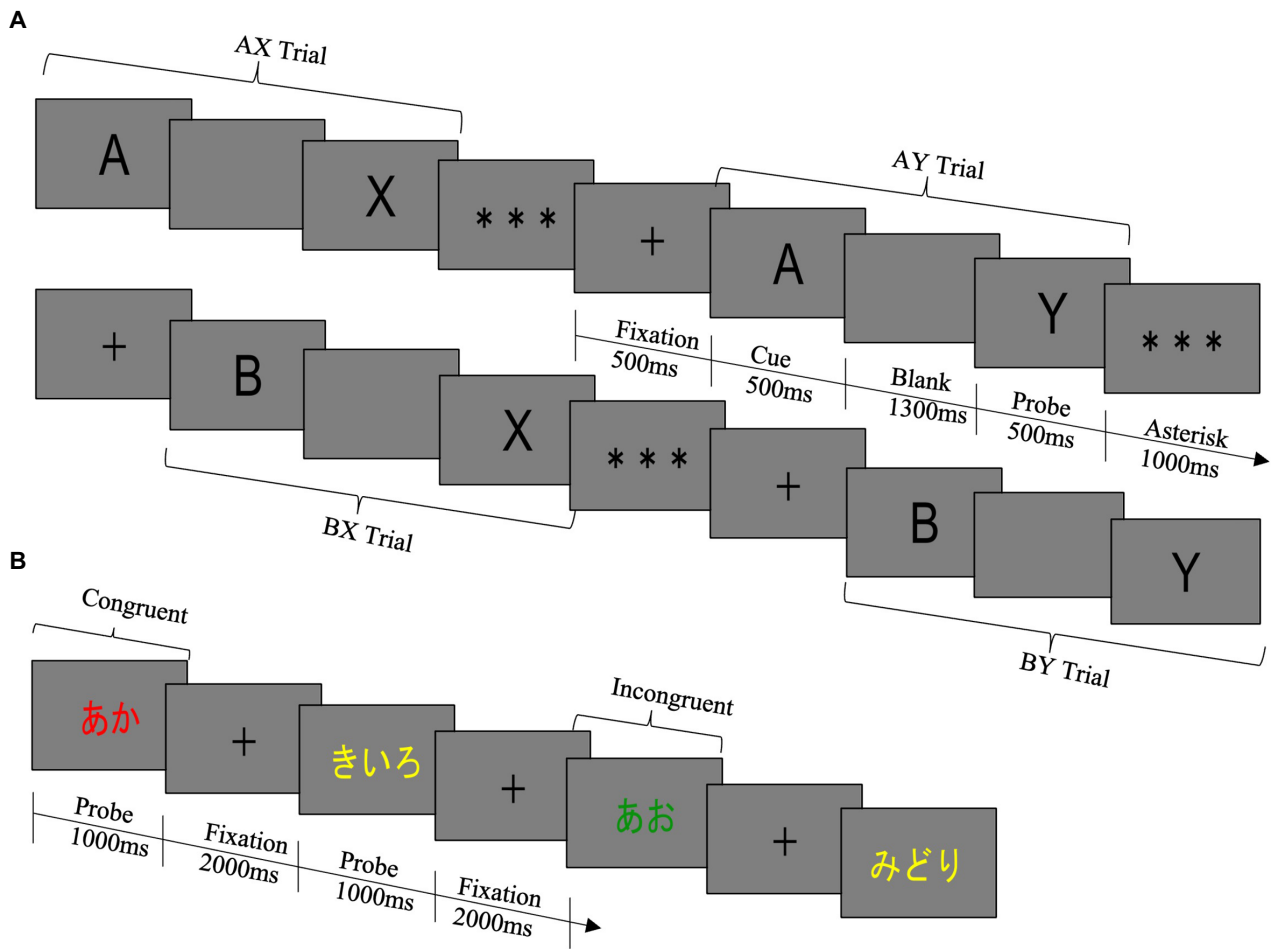
**(A)** AX-CPT. The rule of the task was to press the target key only if A was followed by X. In all other trial types, the non-target key was required to be pressed. The target key was the left arrow key, and the non-target key was the right arrow key. In both the pre- and the post-test, the task was divided into two blocks, and the number of trials in each block was 100 trials. The pre-test consisted of blocks 1 and 2, and the post-test consisted of blocks 3 and 4. Within a block, the proportion of trial types was 70% for AX (true cue-correct probe) trials, 10% for AY (true cue-false probe) trials, 10% for BX (false cue-true probe) trials, and 10% for BY (false cue-false probe) trials, and the order in which each trial type was presented was completely random. In this study, the false cue stimulus and the false probe stimulus were randomly selected from letters except for the true cue stimulus “A” and the true probe stimulus “X.” The same letter was never used in the cue stimulus and the probe stimulus. The same letter was never used for both cue and probe stimuli. **(B)** Modified Stroop Task. The rule was to press the button corresponding to the ink color of the letter: d for red, f for blue, j for green, and k for yellow. The keys were marked with stickers of the corresponding color. In both the pre- and the post-test, the task was divided into three blocks, and the number of trials in each block was 80 trials. The pre-test consisted of blocks 1 to 3, and the post-test consisted of blocks 4 to 6. Within a block, the proportion of trial types was 70% for congruent trials and 30% for incongruent trials and the order in which each trial type was presented was completely random.

The structure of one trial of the AX-CPT was as follows (see Figure 1A). First, (1) a fixation point was presented for 500 ms, and then, (2) a cued stimulus was presented for 500 ms. Then, (3) a blank screen was presented for 1,300 ms, (4) a probe stimulus was presented for 500 ms, and (5) an asterisk was presented for 1,000 ms. Participants responded by pressing keys between the presentation of the probe stimulus and the end of the asterisk presentation.

Error rates and average RTs were recorded separately for each of the four trial types (AX, AY, BX, and BY). Inaccurate and missing responses were used to calculate error rates. RTs were calculated using only the correct responses.

##### Computation of Indices for Control Modes

Three additional indices reflecting tendencies to use proactive control were also computed: the Proactive Behavioral Index (PBI), the *d*’-context, and A-cue bias.

The first index, PBI, was calculated from *[(AY trials − BX trials)/(AY trials + BX trials)]* (Braver et al., 2009; Edwards et al., 2010). This index reflects the relative balance of interference between AY and BX trials such that a positive PBI reflects higher interference on AY trials, indicating proactive control, whereas a negative PBI reflects higher interference on BX trials, indicating reactive control. The PBI was computed separately for error rates and average RTs on AY and BX trials.

The other two indices, *d*’-context and A-cue bias, are based on signal detection theory (Stanislaw and Todorov, 1999). The *d*’-context was calculated from *[Z(AX trial hits) − Z(BX trial errors)]* with Z representing the *z*-transform of a value (Gonthier et al., 2016). These indices reflect the ability of participants to use contextual information from the cue to drive their response on the X probe (e.g., Barch et al., 2001). The A-cue bias was calculated from 1/2*[Z(AX trial hits) + Z(AY trial errors)] (Richmond et al., 2015). This index reflects the tendency for participants to make a target response following an A-cue, independently of the identity of the probe (Gonthier et al., 2016).

These three indices have the advantage of combining information from multiple trial types into one, which can efficiently capture the involvement of proactive control.

Other additional indices reflecting a tendency to use reactive control were also computed. The index of BX probe interference (BPI) was calculated from *[BX trials − BY trials]* (Husa et al., 2021). Participants who tend to use reactive control retrieved cue contexts after the X probe was presented, resulting in increased errors and long RTs in the BX trial, but not in the BY trial. In contrast, participants who tend to use proactive control will perform relatively similarly on BX and BY trials, because they actively maintain contextual information and are prepared to respond quickly and correctly to B cue trials.

In order to correct for trials where error rates were equal to zero, a log-linear correction was applied to all error rate data prior to computing the PBI of error rates, the *d*’-context, the A-cue bias, and the BPI for error rates (as in Braver et al., 2009; see also Hautus, 1995). This correction was applied as: *error rate* = *(number of errors + 0.5)*/*(number of trials + 1)*.

#### Modified Stroop Task Design and Procedures

We followed the modified Stroop task procedure used by Lesh et al. (2013) in the DMC framework. The Stroop task requires an individual to quickly and accurately give the name of a color by looking at a word written in the ink of a certain color (Stroop, 1935). The Stroop task consists of two conditions: congruent trials in which the color and color name match, and incongruent trials in which the color and color name do not match. In the modified Stroop task, the population of incongruent trial is lower than congruent trials, leading participants to reactively inhibit rarely presented incongruent trial (Lesh et al., 2013). Therefore, low error rates and short RTs on incongruent trials indicate reactive control performance. The structure of one trial of the modified Stroop task was as follows (see Figure 1B). First, (1) a fixation point was presented for 2,000 ms, and then (2) a probe stimulus was presented until the participant responded or 1,000 ms elapsed.

### Assignment of Group Conditions for Movement Tasks

Considering the effect of large changes in posture on the execution of cognitive tasks, we selected movement and exercise tasks that could be performed while seated in a chair.

#### Dohsa-hou Shoulder Movement Task in the Treatment Group

The specific procedure of the experimentally manipulated shoulder movement task is depicted in Supplementary Material 1. For the Dohsa movement task, we selected warm-up movements and a shoulder raising and lowering task, referring to the “preparation stage” and “shoulder raising and lowering program” provided by Yamanaka and Tominaga (2000). The shoulder raising and lowering task allows the participants to experience and confirm the sensation of independently moving their bodies, and it is also a movement task that can easily promote a sense of monitoring one’s activity, such as efforts in movement, because the range of the body to be adjusted and controlled on one’s own is wide and tacit movements are likely to appear (Takeuchi, 2016). The duration of each was approximately 4–5 min for the warm-up movement and 6–7 min for the shoulder movement task.

#### Exercise-Based Movement Task in the Active Control Group

The active control movement task was based on the “*undo kadai*” (translated as “exercise task”) used by Takeuchi (2016), and the procedure was repeated three times with the experimenter cueing the participants to keep their shoulders raised for 2 min, and then lower their shoulders for 2 min. During the exercise task, the experimenter did not touch the participant’s body, but interacted with the participant only to announce the beginning and end of the task. The duration was approximately 12 min.

In the Dohsa-hou group, the experimenter communicated with the participants to maintain their attention to their body movements and bodily sensations, whereas in the active control group, the experimenter did not, so the participants did not pay any particular attention to their bodies or reproduced their usual movement patterns. Since the movement of raising and holding the shoulders is not a movement that is always experienced, it was expected that attention would be directed to the body, but the degree and intensity of this attention was expected to be less than that experienced in the Dohsa-hou group.

#### Passive Resting Condition in Passive Control Group

The participants in the passive control group were asked to rest freely for the same amount of time as they performed the exercise task. They were told to avoid sleeping.

### Experimental Procedure

After entering the laboratory, the participants received a written and verbal explanation of the experiment from the experimenter and signed an informed consent form. Then, the participants performed the AX-CPT or the modified Stroop task at pre-test. Next, the participants were assigned to either three groups and performed each task. The participants then performed the same cognitive task as in the pre-test again at post-test. About a week later, the participants performed a different cognitive task from the first day of the experiment using the same procedure. The order in which the cognitive tasks were performed was counterbalanced.

### Statistical Analysis

The software JASP (Version 0.10.2; JASP Team, 2019) was used for statistical analysis. Tests of the hypotheses were performed with *p* < 0.05 as the criterion for statistical significance. The effect sizes and confidence intervals for each test were used to determine whether hypotheses were supported.

#### AX Version of Continuous Performance Test

AX version of Continuous Performance Test error rates and RTs were examined using a 3 (Group: Dohsa-hou group vs. active control group vs. passive control group) × 2 (Test Time: pre-test and post-test) × 4 (Trial Type: AX, AY, BX, and BY) repeated measures analysis of variance (ANOVA). Indices based on proactive and reactive control were examined using a 3 (Group) × 2 (Test Time) repeated measures ANOVA. In the repeated measure ANOVAs, if Mauchly’s test of sphericity assumption was violated, the Greenhouse–Geisser epsilon was used to correct the degrees of freedom. Multiple contrasts were corrected using the Bonferroni method.

Furthermore, for the PBI of error rates and RTs, we used a one-sample *t*-test with the test value equal to zero to indicate individual dominance on cognitive control, in reference to Gonthier et al. (2016) and Chang et al. (2018).

#### Modified Stroop Task

Modified Stroop task error rates and RTs were examined using a 3 (Group) × 2 (Test Time) × 2 (Trial Type: Congruent and Incongruent) repeated measures ANOVA.

## Results

Supplementary Material 2 details the full summaries of descriptive statistics and reliability analysis results for the cognitive tasks.

### AX Version of Continuous Performance Test

The mean values of pre- and post-test error rates and RTs of the three groups of AX-CPT used in the final analysis are shown in Table 1.

**Table 1:** Descriptive statistics of AX-CPT for Dohsa-hou group, Active control group, and Passive control group measured at pre- and post-test.

	Dohsa-hou (*N* = 12)		Active control (*N* = 13)		Passive control (*N* = 26)	
	Pre-test	Post-test	Pre-test	Post-test	Pre-test	Post-test
	Mean (SD)	Mean (SD)	Mean (SD)	Mean (SD)	Mean (SD)	Mean (SD)
*Error rate (%)*						
AX	1.37 (1.62)	1.07 (1.38)	1.76 (1.32)	1.92 (1.76)	2.86 (4.46)	2.77 (2.61)
AY	3.75 (5.69)	5.00 (5.64)	6.15 (6.50)	5.77 (7.87)	6.15 (8.75)	12.31 (11.07)
BX	1.25 (2.26)	3.75 (5.28)	2.69 (4.39)	3.85 (5.83)	4.23 (7.03)	6.15 (7.39)
BY	0.83 (1.95)	0.83 (1.95)	0.77 (1.88)	3.85 (8.93)	2.31 (5.14)	2.88 (5.86)
PBI for error rate	0.14 (0.26)	0.09 (0.45)	0.17 (0.41)	0.12 (0.24)	0.09 (0.41)	0.23 (0.45)
*d’*-context	4.13 (0.44)	4.01 (0.66)	3.86 (0.55)	3.78 (0.60)	3.78 (0.87)	3.50 (0.67)
A-cue bias	0.30 (0.25)	0.38 (0.21)	0.30 (0.30)	0.27 (0.18)	0.27 (0.32)	0.39 (0.25)
BPI for error rate	0.42 (3.34)	2.92 (6.20)	1.92 (4.80)	0.00 (5.40)	1.92 (4.02)	3.27 (4.89)
*RT (ms)*						
AX	473.37 (143.09)	413.82 (116.29)	524.55 (193.08)	477.78 (182.68)	515.01 (164.48)	444.25 (122.89)
AY	547.92 (119.62)	503.76 (84.72)	599.34 (159.94)	568.47 (141.64)	601.50 (142.64)	557.74 (121.77)
BX	445.53 (171.43)	363.60 (148.00)	473.36 (201.98)	408.40 (219.61)	475.68 (187.96)	382.56 (153.98)
BY	441.54 (173.21)	358.50 (160.15)	482.55 (210.46)	418.56 (190.54)	469.78 (179.22)	387.35 (147.59)
PBI for RT	0.13 (0.10)	0.18 (0.10)	0.14 (0.10)	0.20 (0.13)	0.14 (0.10)	0.21 (0.09)
BPI for RT	3.99 (24.74)	5.10 (33.06)	−9.19 (42.83)	−10.16 (46.83)	5.9 (40.27)	−4.79 (48.56)

*N*, number of data used in the analysis; SD, standard deviation.

#### Error Rate

**Figure 2:**
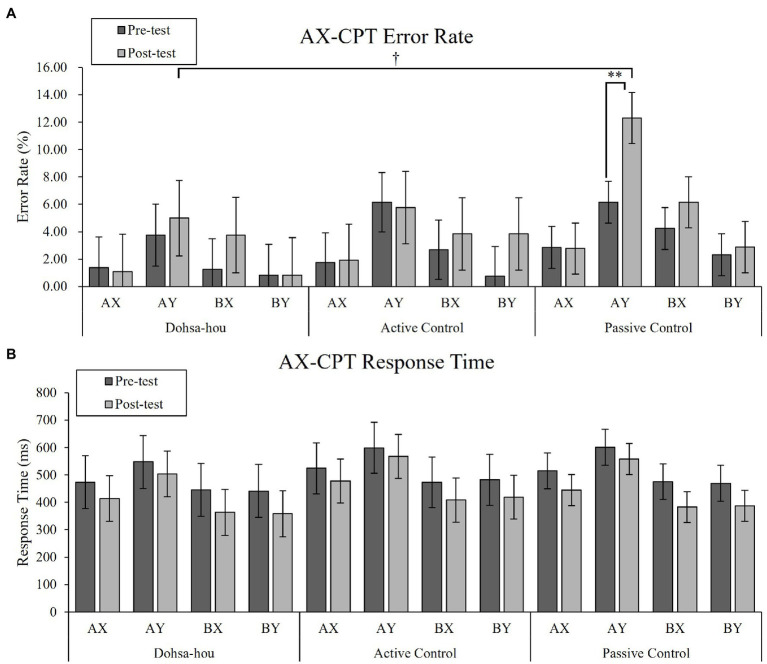
The error bars represent standard error of the mean. **(A)** Mean error rate of the AX-CPT for each group, each test time, and each trial type. **(B)** Mean response time of the AX-CPT for each group, each test time, and each trial type. Asterisks represents a significant difference. ^†^*p* < 0.10; ^**^p < 0.01.

For error rates, the Group (Dohsa-hou, active control, passive control) × Test Time (pre, post) × Trial Type (AX, AY, BX, and BY) repeated measures ANOVA (see Figure 2A) revealed a significant main effect of Trial Type, *F*(1.68, 80.47) = 15.01, *p <* 0.001, *η_p_*^2^ = 0.24, 95% CI [0.09, −0.40]. In addition, there was a marginal three-way interaction, *F*(4.78, 48) = 2.10, *p* = 0.074, *η_p_*^2^ = 0.08, 95% CI [0.00, 0.16]. A simple main effect of Group only on AY trial in the post-test was significant, *F*(2, 48) = 3.54, *p =* 0.037, *η_p_*^2^ = 0.13, 95% CI [0.00, 0.29], indicating error rate of AY trial in the post-test was marginally greater in passive control group than in Dohsa-hou group, *t*(48) = −2.25, *p* = 0.087, *d* = −2.21 95% CI [−3.04, −1.37]. Furthermore, a simple main effect of Test Time only on AY trial in passive control group, *F*(1, 25) = 10.19, *p* = 0.004, *η_p_*^2^ = 0.29, 95% CI [0.04, 0.51], indicating the passive control group showed an increase in error rates in the AY trial from pre- to post-test. This increase was not observed in other groups [in the Dohsa-hou group: *F*(1, 11) = 1.94, *p* = 0.191, *η_p_*^2^ = 0.15, 95% CI (0.00, 0.47), in the active control group: *F*(1, 12) = 0.03, *p* = 0.870, *η_p_*^2^ = 0.00, 95% CI (0.00, 0.19)]. Also, only in the post-test of the passive control group, a simple main effect of Trial Type was significant, *F*(3, 75) = 13.60, *p* < 0.001, *η_p_*^2^ = 0.35, 95% CI [0.17, 0.47], indicating the error rate of the AY trial was higher than in the AX and BY trials (*p*s < 0.002). Both the greater degree of improvement and difference from other trial types for the AY trial are consistent with the increased tendency to use proactive control that was observed only in the passive control group, whose tendency was more pronounced at post-test than the Dohsa-hou group.

#### Response Time

For RTs, the Group × Test Time × Trial Type repeated measures ANOVA (see Figure 2B) revealed a significant two-way interaction between Test Time and Trial Type, *F*(2.30, 110.35) = 9.94, *p* < 0.001, *η_p_*^2^ = 0.17, 95% CI [0.05, 0.28], main effect of Trial Type, *F*(1.62, 77.74) = 113.47, *p* < 0.001, *η_p_*^2^ = 0.70, 95% CI [0.58, 0.77]. In both test times, the simple effect of Trial Type was significant [in pre-test: *F*(3, 144) = 65.00, *p* < 0.001, *η_p_*^2^ = 0.58, 95% CI (0.46, 0.65), in post-test: *F*(3, 144) = 125.42, *p* < 0.001, *η_p_*^2^ = 0.72, 95% CI (0.64, 0.77)], indicating the RTs of the AY trial were longer than the other trial types (*p*s < 0.001), and the RTs of the AX trials were longer than that of BX and BY trial (*p*s < 0.002). In all trial types, the simple main effect of Test Time was significant (*p*s < 0.014), indicating that RTs decreased in all trial types from pre- to post-test. There was no difference in RTs among the groups, and the Trial Type profile was similar in both the pre- and post-test, indicating an overall improvement in performance over time.

#### Indices Based on Proactive Control

For the PBI of error rates, the Group × Test Time repeated measures ANOVA revealed no significant effect (*p* > 0.41). With regard to the PBI of RTs, the Group × Test Time repeated measures ANOVA revealed the main effect of Test Time, *F*(1, 48) = 27.37, *p* < 0.001, *η_p_*^2^ = 0.36, 95% CI [0.15, 0.52], showing that the PBI of RTs increased from pre- to post-test. In addition, a one-sample *t*-test was performed on the PBI of error rates and RTs at both test times for each group. Importantly, only in the Dohsa-hou group, the PBI of error rates was marginally significantly higher than zero in the pre-test [*t*(11) = 1.83, *p* = 0.095, *d* = 0.53, 95% CI (0.01, 1.03)], but the same as zero in the post-test [*t*(11) = 0.70, *p* = 0.502, *d* = 0.20, 95% CI (−0.28, 0.68)]. However, in both the active control group and passive control group, the PBIs of error rates were equal to zero in the pre-test [active control group: *t*(12) = 1.50, *p* = 0.159, *d* = 0.42, 95% CI (−0.01, 1.03), passive control group: *t*(25) = 1.12, *p* = 0.275, *d* = 0.22, 95% CI (−0.11, 0.54)], while they were marginally or significantly higher than zero in the post-test [active control group: *t*(12) = 1.86, *p* = 0.088, *d* = 0.52, 95% CI (0.02, 0.99), passive control group: *t*(25) = 2.67, *p* = 0.013, *d* = 0.52, 95% CI (0.18, 0.86)]. Participants in the Dohsa-hou group exhibited more balanced cognitive control after intervention. The PBI of RTs was significantly greater than zero across test times and groups (*p*s < 0.001), indicating a proactive control dominance.

For *d’-*context, the Group × Test Time repeated measures ANOVA revealed a marginal main effect of Test Time, *F*(1, 48) = 3.74, *p* = 0.059, *η_p_*^2^ = 0.07, 95% CI [0.00, 0.24], indicating the *d’*-context decreased from pre- to post-test. The tendency to use cue information for the “X” probe response decreased regardless of group.

Finally, there was no significant effect on A-cue bias (*p*s > 0.27), reflecting that the tendency to prepare a target response after an A-cue was unaffected by group manipulation or test time.

#### Indices Based on Reactive Control

For the BPI of error rates, the Group × Test Time repeated measures ANOVA revealed no significant effect (*p*s > 0.27). With regard to the BPI of RTs, the Group × Test Time repeated measures ANOVA revealed a significant main effect of Test Time [*F*(1, 48) = 4.29, *p* = 0.044, *η_p_*^2^ = 0.04, 95% CI (0.00, 0.25)], indicating that scores for the BPI of RTs decreased from pre- to post-test. The difference in RTs between BX and BY trials tended to decrease from pre- to post-test.

### Modified Stroop Task

There were no trials where the response time was less than 200 ms, and no exclusions were made for each trial. The mean values of pre- and post-test error rates and RTs of the three groups of modified Stroop Task used in the final analysis are shown in Table 2.

**Table 2:** Descriptive statistics of Modified Stroop Task for the Dohsa-hou group, Active control group, and Passive control group measured at pre- and post-test.

	Dohsa-hou (*N* = 13)		Active control (*N* = 12)		Passive control (*N* = 28)	
	Pre-test	Post-test	Pre-test	Post-test	Pre-test	Post-test
	Mean (SD)	Mean (SD)	Mean (SD)	Mean (SD)	Mean (SD)	Mean (SD)
*Error rate (%)*						
Congruent	3.53 (3.78)	3.21 (2.92)	3.97 (4.78)	4.12 (4.10)	5.65 (6.41)	5.10 (5.93)
Incongruent	9.94 (8.45)	7.16 (5.00)	13.08 (7.73)	11.57 (6.63)	11.36 (7.27)	9.37 (7.29)
*RT (ms)*						
Congruent	558.85 (57.81)	550.26 (55.44)	544.56 (40.04)	536.91 (64.68)	560.47 (66.24)	547.07 (65.72)
Incongruent	608.38 (64.53)	597.33 (70.16)	609.58 (41.84)	600.32 (61.05)	631.73 (73.45)	599.24 (66.58)

*N*, Number of data used in the analysis; SD, standard deviation.

#### Error Rate

**Figure 3:**
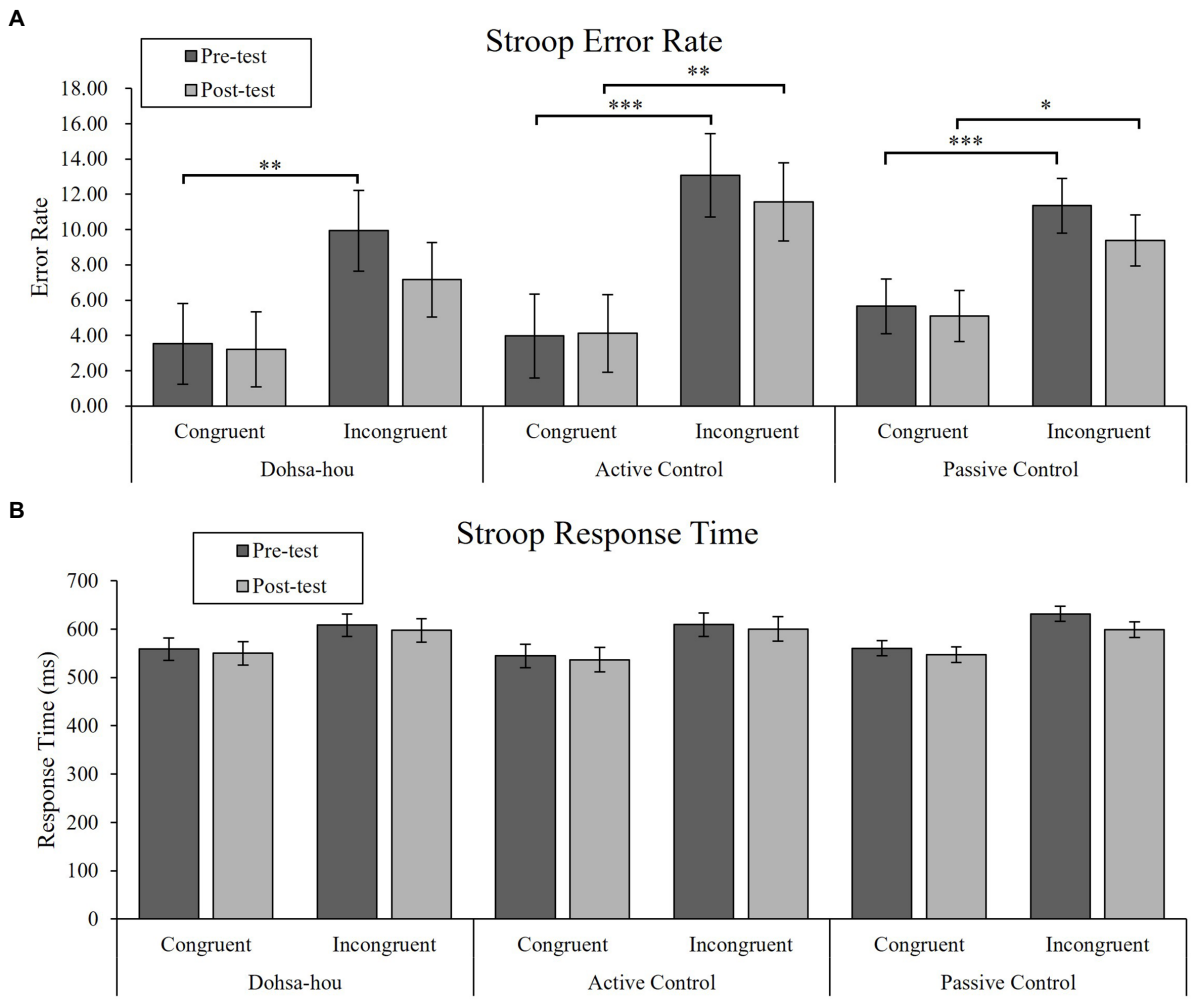
The error bars represent standard error of the mean. **(A)** Mean error rate of the Modified Stroop Task for each group, each test time, and each trial type. **(B)** Mean response time of the Modified Stroop Task for each group, each test time, and each trial type. Asterisks represents a significant difference. ^∗^*p* < 0.05; ^∗∗^*p* < 0.01; ^∗∗∗^*p* < 0.001.

For error rates, the Group (Dohsa-hou, active control, passive control) × Test Time (pre, post) × Trial Type (congruent, incongruent) repeated measures ANOVA (see Figure 3A) revealed a significant two-way interaction between Test Time and Trial Type [*F*(1, 50) = 5.08, *p* = 0.029, *η_p_*^2^ = 0.09, 95% CI (0.00, 0.26)], main effect of Test Time [*F*(1, 50) = 4.28, *p* = 0.044, *η_p_*^2^ = 0.08, 95% CI (0.00, 0.24)], and Trial Type [*F*(1, 50) = 70.11, *p* < 0.001, *η_p_*^2^ = 0.58, 95% CI (0.39, 0.70)]. Further analysis showed that the error rate for incongruent trials only decreased from pre- to post-test [*F*(1, 50) = 5.91, *p* = 0.019, *η_p_*^2^ = 0.11, 95% CI (0.00, 0.27)]. In addition, the error rate for incongruent trials was higher than for congruent trials at both test times [pre-test: *F*(1, 50) = 52.60, *p* < 0.001, *η_p_*^2^ = 0.51, 95% CI (0.31, 0.64), post-test: *F*(1, 50) = 58.91, *p* < 0.001, *η_p_*^2^ = 0.54, 95% CI (0.34, 0.66)], indicating the appearance of a consistent Stroop effect. We conducted planned comparisons to determine differences between and within groups. There were no differences (*p*s > 0.31). However, only the Dohsa-hou group showed a significant difference between congruent and incongruent trials in the pre-test [*t*(100) = −4.05, *p* < 0.001, *d* = −1.46, 95% CI (−2.57, −0.36)], but not in the post-test [*t*(100) = −2.50, *p* = 0.958, *d* = −0.65, 95% CI (−1.42, 0.11)]. This indicated that the Stroop effect disappeared, and that reactive control was optimized. For the other groups, the Stroop effect was present at both test times [active control group at pre-test: *t*(100) = −5.54, *p* < 0.001, *d* = −1.51, 95% CI (−2.40, −0.61), at post-test: *t*(100) = −4.53, *p <* 0.001, *d* = −1.23, 95% CI (−2.08, −0.38), passive control at pre-test: *t*(100) = −5.29, *p* < 0.001, *d* = −0.94, 95% CI (−1.48, −0.41), at post-test: *t*(100) = −4.00, *p* < 0.001, *d* = −0.72, 95% CI (−1.25, −0.19)].

#### Response Time

For RTs, the Group × Test Time × Trial Type repeated measures ANOVA (see Figure 3B) revealed a significant main effect of Test Time [*F*(1, 50) = 8.00, *p* = 0.007, *η_p_*^2^ = 0.14, 95% CI (0.01, 0.31)], and Trial Type [*F*(1, 50) = 114.86, *p* < 0.001, *η_p_*^2^ = 0.70, 95% CI (0.54, 0.78)]. In addition, there was a marginal two-way interaction between Test Time and Trial Type [*F*(1, 50) = 3.32, *p* = 0.075, *η_p_*^2^ = 0.06, 95% CI (0.00, 0.22)]. Further analysis showed that RTs for both trial types decreased from pre- to post-test [for congruent trial: *F*(1, 50) = 4.46, *p* = 0.040, *η_p_*^2^ = 0.08, 95% CI (0.00, 0.24), for the incongruent trial: *F*(1, 50) = 9.03, *p* = 0.004, *η_p_*^2^ = 0.15, 95% CI (0.02, 0.33)], and that RTs for incongruent trials were higher than for congruent trials at both test times [pre-test: *F*(1, 50) = 122.65, *p* < 0.001, *η_p_*^2^ = 0.71, 95% CI (0.56, 0.79), post-test: *F*(1, 50) = 80.67, *p* < 0.001, *η_p_*^2^ = 0.62, 95% CI (0.43, 0.72)].

## Discussion

The aim of this study was to examine the empirical basis for the types of control modes and performance of cognitive control after exposure to the shoulder movement task used in the psychotherapy, Dohsa-hou, compared to control conditions. Our study proposed three hypotheses about the performance of proactive control *via* AX-CPT and reactive control *via* the modified Stroop task, such that the Dohsa-hou treatment group would demonstrate a shift to the reactive mode and indicators of flexible cognitive control performance. Each of these hypotheses is evaluated with the key findings in the sections that follow. We first evaluated the results against the findings of previous studies in terms of reported descriptive statistics, reliability, and computed task paradigm-related indices that reflect features of cognitive control.

### Comparison of Descriptive Statistics, Reliability Analyses, and Computed Indices

Descriptive statistics are depicted in Tables 1 and 2. For the AX-CPT, the mean value of RTs at pre-test was slightly longer than that of previous studies (e.g., Gonthier et al., 2016; Li et al., 2018). Therefore, the RTs of all trial types improved from the pre- to the post-test, but this may be due to the practice effect of repeated use of the task, rather than a shift in the control mode, which reduced RTs. Also, Cooper et al. (2017) noted the difficulty in interpreting RTs as reflecting not only the cognitive demands of a given condition or trial type, but also other individual difference components, such as processing speed. Thus, we preferred the interpretation of error rates over RTs. For the modified Stroop task, the mean value of RTs in the pre-test was slightly shorter than that of healthy control participants reported by Lesh et al. (2013). We considered that the RTs of the modified Stroop task are also affected by individual differences, such as processing speed, so we prioritized the interpretation of error rates over RTs.

The procedure for analysis of reliability with psychometric theory was based on Cooper et al. (2017) and performed for the blocks of trials with intraclass correlation coefficients (ICCs) at pre-test and post-test. Like the detailed reliability analyses of their study, the trials showed varying levels of reliability in terms of Cronbach’s alpha (Cooper et al., 2017). The AX trials were the most consistent at pre-test (*α* = 0.87) and post-test (*α* = 0.71), followed by AY (*α* = 0.57; *α* = 0.61), BX (*α* = 0.57, *α* = 0.47), and BY (*α* = 0.54, *α* = 0.71). Intraclass correlation coefficients by trial type generally supported reliability for all pre-test blocks 1–2 (ICCs = 0.40–92), except for BY (ICC = 0.24), and blocks 3–4 at post-test (ICCs = 0.44–0.65), showing strengths and weaknesses in terms of variance as stated in the previous study (Cooper et al., 2017). For the purposes of our study, we considered the majority of these task reliability indices as comparable to those previously evaluated with psychometric theory (Cooper et al., 2017). See Supplementary Material 2 for full details on reliability analyses.

We computed other main indices in the DMC framework that reflect the dynamics of control modes as instructed by previous studies. Calculations for the PBI, *d*’-context, and A-cue bias were performed according to Gonthier et al. (2016), and the BPI according to Husa et al. (2021). The PBI results are discussed in detail for the following sections, but one finding that we did not anticipate emerged for the PBI of RTs. In contrast to Li et al. (2018), our results for the PBI of RTs were numerically higher for all conditions. Despite these differences, comparison of standard errors with theirs suggests overlap with our results. Nevertheless, Cooper et al. (2017) determined that PBI of RTs might be difficult to draw conclusions about in terms of reliability and task construct representation due to the need for more *a priori* studies using the performance measure. This issue was also mentioned for signal detection theory-based indices like *d’*-context (Cooper et al., 2017). Our results for *d’-*context suggested no differences between group conditions, however, as the DMC framework does not make strong predictions about the role of *d’*-context, we have chosen not to focus on those results nor the PBI of RTs for the present report but offer them for evaluation by AX-CPT researchers.

Cumulatively, the results suggested that the reliability of the study variables were comparable to known parameters in previous studies, and that mean levels of AX-CPT error rates, modified Stroop task error rates, and the main DMC framework indices of the PBI for error rate, BPI for error rate, and A-cue bias were interpretable for the aims of our study. Interpretations of our means-based comparisons for the groups in synthesis with Dohsa-hou research are discussed against our hypotheses in the following sections.

### Key Findings for Proactive Control in the AX-CPT: Hypotheses 1 and 2

Our first hypothesis (H1) was that exposure to the Dohsa-hou movement tasks would be less affiliated with the proactive control mode. We focused on repeated measures ANOVA for the error rates of each trial in AX-CPT to examine the performance of proactive control. Key findings emerged for the AY trial results. In the passive control group, the error rate of AY trials increased from pre- to post-test, indicating that repeated practice of AX-CPT enhances proactive control. This is supported by the fact that A-cue bias, which indicates a tendency to prepare a target response based on A-cue, showed a greater increase from 0.27 to 0.39 in the passive control group, although there was no statistical difference. However, these numerical increases in the error rate of AY trials were not observed in the Dohsa-hou group nor the active control group, and we determined that possible practice effects were counteracted. The confidence intervals for effect sizes in the Dohsa-hou and active control groups included zero, but not in the passive control group, in favor a statistically significant difference. Particularly, the error rate of AY trials in the post-test of Dohsa-hou group was lower than that of the passive control group, but the difference was only marginally statistically significant, therefore refuting H1. Together, these findings indicate that moving the body or paying attention to body movements in Dohsa-hou may relax the reinforcement of proactive control from repeated implementation of the AX-CPT.

Our second hypothesis (H2) was that exposure to the Dohsa-hou movement task would induce flexible cognitive control performance. We focused on the results of the repeated measures ANOVA and one-sample *t-*test for the PBI for error rate to examine bias toward proactive control. The PBI for error rates combining AY and BX trials reflects the dominance of the control modes (Braver et al., 2009). There were no statistically significant differences between and within groups. However, the PBI was not different from zero at post-test for the Dohsa-hou group only, whereas it was significantly greater than zero in the other two groups in post-test. The confidence interval for the effect size also includes 0 in the Dohsa-hou group, but not for the other groups in post-test, supporting these statistical differences.

These results support H2 and suggest that Dohsa-hou prevents bias toward proactive control and promotes flexible cognitive control. These results are similar to Chang et al. (2018), who examined the effects of mindfulness on both reactive and proactive control modes. By extension, the results suggest that the self-regulatory mechanism of attention in Dohsa-hou might be comparable to the flexible performance of cognitive control found in exposure to mindfulness interventions.

Previous research on the development of cognitive control has indicated that proactive control increases and reactive control decreases relatively throughout growth from childhood (Munakata et al., 2012; Gonthier et al., 2019). The proactive control mode has the benefit of optimizing cognitive processing toward goal activation and maintenance, as goals are activated and retained prior to control. However, it also has the cost of consuming more attentional resources and neglecting the processing of bottom-up information unrelated to the goal. In other words, too much bias toward proactive control can have the consequence of inhibiting learning and imaginative problem-solving (Amer et al., 2016). In Dohsa-hou, the client is made to exert effort toward a body movement as a targeted clinical task but is also asked to continuously monitor and flexibly modify body movements through communication with the therapist. Specifically, verbal instruction for attention to body movement execution and guidance in performance by touch with the hands. In this dynamic where already learned motor patterns are repeated for a purpose, it is possible that some of the proactive control aspects of the body movement might be mitigated. The reason that the PBI feature of the error rate observed in the Dohsa-hou group was not observed in the active control group might be that attentional guidance from the therapist in Dohsa-hou is the key to influencing the control mode. In other words, the therapist’s attentional guidance may facilitate the client’s process of paying attention to, monitoring, and controlling his or her own body movement in a goal-dependent or goal-oriented process.

In favor of this process, we observed a shift toward the reactive mode in the Dohsa-hou group from the AX-CPT results. However, due to the experimental limitations of specificity of the AX-CPT paradigm, it is difficult to determine whether the relaxation of proactive control in Dohsa-hou is synonymous with the facilitation of reactive control. The BPI for error rates an indicator of reactive control in AX-CPT did not show any difference between groups and within groups. This indicates that changes in reactive control could not be detected in AX-CPT. This has also been observed in a previous study that noted that, because the AX-CPT is a task that requires sequential processing, measures of reactive control can be confounded by the extent to which processes of proactive control are involved (Lesh et al., 2013). The modified Stroop task has a larger proportion of congruent trials relative to incongruent trials, which relaxes the rule maintenance of the Stroop task itself, requiring participants to exercise reactive inhibition on the rare incongruent trials presented. In other words, this task allows us to assess the performance of reactive control while relaxing proactive control (e.g., Yang et al., 2018). Therefore, for this interpretation of the data, we looked to the strengths of the alternative experimental paradigm (modified Stroop task) for inferences as it extracts reactive control only, as detailed in the following section.

### Key Findings for Reactive Control in the Modified Stroop Task: Hypothesis 3

Our third hypothesis (H3) was that exposure to the Dohsa-hou movement tasks would increase the reactive control mode. We focused on repeated measures ANOVA for error rates of the modified Stroop task to examine the performance of reactive control. Key findings emerged for the Stroop effect in terms of the difference between congruent trial and incongruent trial. Our results showed that regardless of group, the error rates of incongruent trials decreased from pre- to post-test, indicating an improvement in reactive control. Although the error rate of incongruent trials in Dohsa-hou group changed numerically from 9.94 to 7.16%, which was the larger decrease compared to other groups (see Table 2), planned comparisons failed to reveal differences between groups. However, another planned comparison revealed that the error rate of incongruent trials approached that of congruent trials to such an extent that the Stroop effect was no longer confirmed at post-test, specifically in the Dohsa-hou group only, while the other two groups showed a Stroop effect at both test times. The confidence interval for the effect size includes 0 in the Dohsa-hou group but not in the other groups in post-test, supporting these statistical differences. Taken together, and while relatively modest, we believe the results partially support H3 in suggesting that Dohsa-hou may facilitate reactive control. We believe that this shift might reflect the learning process of body movements in the psychotherapy. In Dohsa-hou, clients experience a new way of controlling their body movements by deliberately monitoring internal and external stimuli with guidance from the therapist as they perform the movement task intentionally by themselves. This approach to modifying and controlling body movements based on movement as a source of evaluation is the aspect of reactive control of movements attributable to Dohsa-hou as an experiencing process. In other words, the partial support for H3 suggests that clients may strengthen their reactive control through the experiential approach found in Dohsa-hou. Typically, reactive control is thought to be induced by anxiety and interpreted as a negative consequence of it (Yang et al., 2018), but as mentioned earlier, the reactive control mode is also characterized by the need to apply bottom-up information from the environment to support learning and imaginative problem-solving. This is further supported by the observation that reactive control becomes de-emphasized throughout development as a process of maturation, which would reinforce tendencies toward inflexible cognitive control performance through habit formation processes. This interpretation of the findings is supported by the role of proprioceptive sense in automatic behavior and acquiring new skills about the body (Liutsko, 2013), especially aligning with the levels of behavioral control by Corr (2010) and descriptions of the phenomenon of late error detection for interrupting routines for adaptive feedback processing (as cited in Tous-Ral et al., 2012; Liutsko, 2013). Therefore, Dohsa-hou may work by breaking through this fixed pattern of cognitive control in support of a more balanced state of self-control.

### Implications for Dohsa-hou Psychotherapists and Meditative Movement Practitioners in Applied Settings

The evidence provided here for a balancing effect from Dohsa-hou movement tasks given in a brief session format indicates that the approach promotes a form of continuous self-monitoring while seated that might reduce mind-wandering for clients. Our findings suggest that this could be attributable to the focus on movement execution from the instructions and feedback with the therapist. In addition, Dohsa-hou has been utilized in the treatment schemes of emotional disorders and body-mediated communication for individuals with developmental conditions. Thus, it might be the case that the control mode shift from guided attention to Dohsa-hou movement tasks is an opportunity for therapists to intervene in the automaticity of behavioral patterns that emerge in clinical settings. An example might include offering movement tasks to disrupt fixed behavioral patterns in autism, or in other clinical presentations. Such a case was described in a clinical report by Fujino (2017) for clients with Down syndrome exhibiting maladaptive behaviors and internalizing symptoms, whose severity was lessened with Dohsa-hou. As reviewed by Kabir et al. (2018), for another case, self-active relaxation tasks based in Dohsa-hou were used as a form of behavioral activation in a case report of a client with depression. Haramaki et al. (2019) also observed an *in vivo* exposure-based ability for long-term hemodialysis patients to address issues with self-regulatory fatigue from chronic pain by using Dohsa-hou tasks. Furthermore, in stress management applications of Dohsa-hou, Abe et al. (2019) observed changes in mood states, especially vigor, in a sample of nurses at-risk for burnout. The role of movement is central to this attentional form of self-regulation and might extend to meditative movement practices with similar elements (e.g., qigong, yoga, or tai chi in applied contexts of rehabilitation or behavioral change). In this way, the opportunity to have clients engage in the movement tasks might provide practitioners in applied settings with a flexible tool for creative problem-solving and state-based change in a transdiagnostic fashion, similar to progressive muscle relaxation. As implications, practitioners might consider the technique for clients to pay attention to body movements in a way that promotes the reactive control mode and relax the proactive control mode and emphasizes its practical transfer to the client’s daily life habits.

### Areas for Future Research

Examinations of the practice and duration effects of Dohsa-hou-based attentional shifts are a major area of further research. These might include cross-sectional comparisons between those with and without the practice of Dohsa-hou or comparisons of longitudinal changes from continuous practice. This is a key test that was conducted in an 8-week mindfulness-based intervention, as an example, which observed a shift from the reactive to the proactive mode that was associated with learning the behavioral practice (Chang et al., 2018). An analysis of individual differences in these attentional shifts with established theories and assessments of interoceptive attention tendencies (Mehling et al., 2012; Kabir, 2019) with empirical correlations to neuroimaging (Stern et al., 2017), body awareness (Mehling et al., 2009; Fujino, 2019), sense of body ownership (Kamikura, 2017), other Dohsa-hou measurement tools (Takeuchi, 2017; Kuwashima et al., 2021) or against other meditative movement approaches as conditions is warranted to further specify effects available to self-report with the DMC and AX-CPT as a behavioral paradigm. In addition, the implications of using the DMC framework to interpret our results might include the role of proactive and reactive control modes in Dohsa-hou applications for clients suffering from schizophrenia (Kamikura and Shimizu, 2021), as the experimental paradigms for the tradeoffs were validated against normative data from individuals with schizophrenia (Edwards et al., 2010; Lesh et al., 2013). In addition, while we used a shoulder movement task as a pragmatic starting point for experimentation with our methodology, representing the mechanism of attentional change from feedback with the therapist, it could be the case that other movements in the Dohsa-hou motor vocabulary might illustrate effects on cognitive control. Other empirically supported Dohsa-hou tasks, such as *kukan no hineri*, or the torso twist (Kabir et al., 2018; Kuwashima et al., 2021), might be fruitful to examine dose–response relationships for self-control or relaxation from Dohsa-hou in future studies. Additionally, studies of the DMC with other modalities of meditative movement, such as qigong, tai chi (e.g., Yeung et al., 2018), or yoga (e.g., Gothe and McAuley, 2015) might provide further basis of comparison or synthesis among approaches in terms of motor action planning attuned to sensory information (Mehling et al., 2009; Tous-Ral et al., 2012).

### Limitations

This study provided new empirical evidence for the effects of Dohsa-hou and control condition movement tasks on indices reflecting cognitive control, however, it has some limitations. This study was not sufficiently blinded to the order of group assignment, and the experimenter’s expectations may have influenced the results. Double-blinding procedures, such as presenting the intervention as a video and presenting it randomly, are needed for further experimental rigor. Additionally, the results for error rates and RTs were different from Chang et al. (2018) and Li et al. (2018), suggesting caution when comparing mean rates in a one-to-one fashion. Further psychometric research on the AX-CPT and modified Stroop tasks would be clarifying as well, especially in the cultural context of Japan, to which our study appears to be one of the few so far to operationalize the DMC framework at this level of detail to internal validity for the tasks. The sample size of two of our group conditions were relatively modest, so in addition to the need for longitudinal studies like Chang et al. (2018), future studies will be needed to examine the effects in a larger sample. Generalizability is also difficult due to the makeup of our sample as only consisting of Japanese participants, who have culturally shaped tendencies that are well-documented (e.g., Butler et al., 2007). Although the psychotherapy of Dohsa-hou has been explored in many different countries and contexts, the overrepresentation of Japanese participants in studies of Dohsa-hou remains an issue and limitation but might also serve as an opportunity for future collaborative research outside of Japan. Online versions of stress management applications of the relaxation movement tasks or online Dohsa-hou for pandemic stress management (Kamikura and Shimizu, 2021) might open the practice up to more diverse populations. The authors will make efforts to make the standardized guided attention task used in the experiment available in English for scholars of cognitive control in order to coordinate replication effect sizes in future studies, such as task battery validation projects on the DMC framework (Braver et al., 2021; Tang et al., 2021).

## Conclusion

This study elucidated the type of inhibitory function that is promoted in the guided attention to movement in Dohsa-hou. Our findings suggest that a specificity for the effect of the shoulder movement task occurs *via* the reactive mode in the DMC framework. We also observed that the Dohsa-hou movement task promoted flexible performance of cognitive control. Our evidence of a balancing effect for a Dohsa-hou-based movement task given in the brief session format indicates a form of continuous self-monitoring while seated. It also suggests that conducting the task might reduce mind-wandering as a result of a client’s focus on movement execution and feedback with the therapist. Together, these findings enhance comparisons with mindfulness and other psychotherapeutic techniques used in clinical settings in terms of their impact on cognitive control. Our results suggest that we might consider the self-regulatory mechanism promoted in clinical Dohsa-hou as one that emphasizes guided shifts in attention through the reactive mode toward more flexibility in cognitive control performance. As implications, practitioners might consider the technique with others in their toolbox of ways to shift cognitive modes and break up fixed behavioral patterns related to body movement as a means to offer a concrete coping skill for the daily lives of clients.

## Data Availability Statement

The original contributions presented in the study are included in the article/Supplementary Material, further inquiries can be directed to the corresponding author.

## Ethics Statement

The studies involving human participants were reviewed and approved by the ethical review committee of the Graduate School of Humanities and Social Sciences, Hiroshima University (No. 2019050). The patients/participants provided their written informed consent to participate in this study.

## Author Contributions

TF contributed to study design, experimental materials, data collection, original draft writing, methodology, software, formal analysis, and revisions. RK provided original draft writing, editing, conceptualization, methodology, formal analysis, and revisions. YH provided study design, project administration, funding acquisition, software, formal analysis, and revisions. All authors read and approved the version for publication.

## Funding

This research was supported in part by an alumni association grant (“The 13th Dream Challenge Award”) from Hiroshima University awarded to TF, as well as in part by JSPS KAKENHI No. 20K20865 awarded to YH in the Program in Psychology, Graduate School of Humanities and Social Sciences, Hiroshima University, Japan. In addition, publication fee support was made possible by basic faculty research funds allocated by Hiroshima University to RK.

## Conflict of Interest

The authors declare that the research was conducted in the absence of any commercial or financial relationships that could be construed as a potential conflict of interest.

## Publisher’s Note

All claims expressed in this article are solely those of the authors and do not necessarily represent those of their affiliated organizations, or those of the publisher, the editors and the reviewers. Any product that may be evaluated in this article, or claim that may be made by its manufacturer, is not guaranteed or endorsed by the publisher.
