# Supplementary material for: Experimental Manipulation of Guided Attention to the Shoulder Movement Task in Clinical Dohsa-hou Induces Shifts in the Reactive Mode and Indicates Flexible Cognitive Control Performance
Source: Front Psychol. 2022 Apr 6;13:785385. doi: 10.3389/fpsyg.2022.785385 (PMC9018985; doi:10.3389/fpsyg.2022.785385)
Supplement: Supplementary file 1 [file Data_Sheet_1.docx]

Supplementary Material

In this section, we report details about the Dohsa-hou movement task and full summaries of the descriptive statistics (mean, variance, minimum, maximum, skewness, and kurtosis) for each group (Dohsa-hou group, Active control group, Passive control group) and each block of AX-CPT and modified Stroop Task. The psychometric characteristics of the study variables were examined in reference to Cooper and colleagues (2017). Alpha coefficients as reliability and intraclass correlation coefficients as internal consistency (ICC; ICC2k) among blocks for each cognitive task were estimated for error rates. In the AX-CPT, we calculated ICCs for Block 1 and 2, which constitute the pre-test, and Block 3 and 4, which constitute the post-test. Similarly, in the modified Stroop task, ICCs were calculated for Block 1, 2 and 3, which constitute the pre-test, and Block 4, 5 and 5, which constitute the post-test. Alpha coefficients and ICCs were calculated using the *psych* package in R (Revelle, 2021).

References:

Cooper, S. R., Gonthier, C., Barch, D. M., & Braver, T. S. (2017). The role of psychometrics in individual differences research in cognition: A case study of the AX-CPT. *Frontiers in Psychology*. 8: 1482. doi: 10.3389/fpsyg.2017.01482

Revelle, W. (2021). psych: Procedures for Psychological, Psychometric, and Personality Research. Northwestern University, Evanston, Illinois. R package version 2.1.9, https://CRAN.R-project.org/package=psych.

# Supplementary Material 1: The Dohsa-hou movement task

As described in the manuscript, for the Dohsa-hou movement task, we selected warm-up movements and a shoulder raising and lowering task, referring to the “preparation stage” and “shoulder raising and lowering program” of Yamanaka and Tominaga (2000). All tasks were done while sitting in a chair.

## *Warm-up Movements*

The warm-up movements consisted of (1) stretching, (2) a resting posture, and (3) a straightened posture, and were performed as preparation for the shoulder raising and lowering task. In (1) stretching, the participants folded their hands and pulled their wrists, elbows, and shoulders up above their heads, while at the same time stretching their legs and putting strength into their entire body. Then, they relaxed. In (2) the resting posture, the participants were asked to adopt a free posture that they thought was relaxing. In (3) the straightened posture, participants were verbally instructed to sit shallowly in the chair with their backs facing away from the backrest and their arms hanging down beside their bodies to straighten their body axes. They were then asked to tilt back and forth, left and right, to find the position where they felt most comfortable sitting and stop there. They repeated the sequence of (2) and (3) three times each, respectively.

# *Shoulder Raising and Lowering Task*

The shoulder raising and lowering task was performed in the posture (3) of warm-up movements. At the experimenter's signal, the participants raised both shoulders so that they were reaching both ears (from standard to tension). When they were unable to raise their shoulders any higher, the experimenter instructed them to check their body condition under the experimenter's instruction. Afterwards, the participants lowered their shoulders at the experimenter’s signal (from tension to relax) and felt their bodies relax. The above series of movements was performed as one set, and three sets were performed. This is illustrated in **Supplementary Figure 1** below.


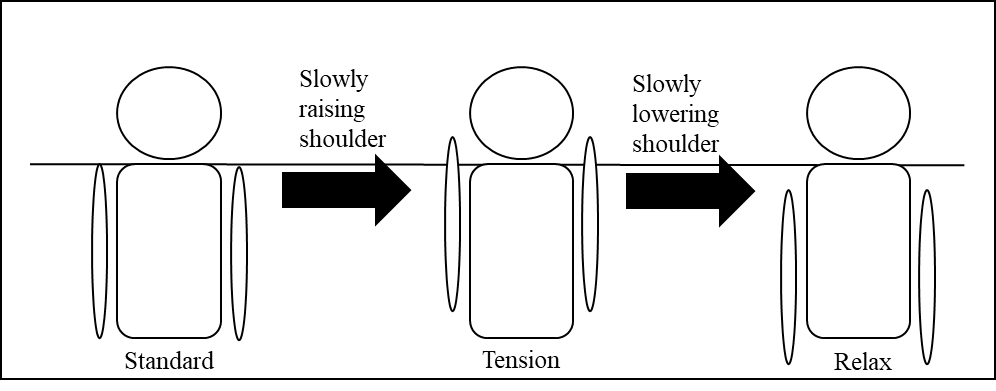


**Supplementary Figure 1.** Shoulder raising and lowering task.

The experimenter instructed the participants to perform this task with their eyes closed as much as possible so that they could easily pay attention to their body movements. Throughout, the experimenter provided verbal instruction and physical prompts by using hands to help participants guide attention to their body movements and bodily sensations, and to independently check and correct their body movements.

In the process of raising the shoulders (from standard to tension), participants were given the verbal instructions, “Let's experience the feeling of strength going into the shoulders.” When the participants lifted their shoulders as far as they could, the experimenter instructed them to pay attention to their face, elbows, hands, waist, and other body parts in turn, and to relax their overexerting.

In addition, in the process of lowering the shoulders (from tension to relax), the experimenter instructed, “Let's experience the feeling of relaxation.” The experimenter also asked the participants, “How does your body feel?” to encourage verbal responses.

We prepared a common manual for the experimenter in advance, so that the same expressions and questions could be asked in manner that was standardized as much as possible.

## Supplementary Material 2: Detailed descriptive statistics

**Supplementary Table 1.** Descriptive statistics for each group on the error rates of the AX-CPT.

| Group | Block | Trail type | Mean ER | Variance | Min | Max | Skew | Kurtosis |
| --- | --- | --- | --- | --- | --- | --- | --- | --- |
| All | Block 1 | AX | 2.32 | 0.11 | 0.00 | 17.14 | 2.45 | 7.64 |
|  |  | AY | 3.53 | 0.51 | 0.00 | 30.00 | 2.41 | 6.06 |
|  |  | BX | 2.16 | 0.33 | 0.00 | 30.00 | 3.23 | 11.62 |
|  |  | BY | 1.57 | 0.25 | 0.00 | 30.00 | 4.17 | 20.38 |
|  | Block 2 | AX | 2.13 | 0.13 | 0.00 | 20.00 | 3.30 | 12.98 |
|  |  | AY | 7.65 | 1.26 | 0.00 | 50.00 | 1.89 | 3.91 |
|  |  | BX | 4.12 | 0.69 | 0.00 | 40.00 | 2.60 | 7.59 |
|  |  | BY | 1.57 | 0.29 | 0.00 | 30.00 | 4.01 | 17.15 |
|  | Block 1-2 | AX | 2.23 | 0.11 | 0.00 | 18.57 | 3.20 | 12.39 |
|  | (Pre-test) | AY | 5.59 | 0.57 | 0.00 | 30.00 | 1.48 | 1.83 |
|  |  | BX | 3.14 | 0.32 | 0.00 | 25.00 | 2.26 | 5.07 |
|  |  | BY | 1.57 | 0.15 | 0.00 | 20.00 | 3.21 | 11.31 |
|  | Block 3 | AX | 2.02 | 0.05 | 0.00 | 11.43 | 1.91 | 5.49 |
|  |  | AY | 7.25 | 1.12 | 0.00 | 50.00 | 2.05 | 5.31 |
|  |  | BX | 4.51 | 0.65 | 0.00 | 30.00 | 1.82 | 2.63 |
|  |  | BY | 2.94 | 0.61 | 0.00 | 40.00 | 3.34 | 12.03 |
|  | Block 4 | AX | 2.30 | 0.09 | 0.00 | 15.71 | 2.24 | 6.90 |
|  |  | AY | 10.59 | 1.46 | 0.00 | 40.00 | 1.02 | 0.16 |
|  |  | BX | 5.49 | 0.69 | 0.00 | 40.00 | 2.11 | 5.76 |
|  |  | BY | 2.35 | 0.42 | 0.00 | 30.00 | 3.35 | 11.78 |
|  | Block 3-4 | AX | 2.16 | 0.05 | 0.00 | 10.71 | 1.76 | 3.70 |
|  | (Post-test) | AY | 8.92 | 0.95 | 0.00 | 45.00 | 1.49 | 2.47 |
|  |  | BX | 5.00 | 0.43 | 0.00 | 25.00 | 1.44 | 1.68 |
|  |  | BY | 2.65 | 0.38 | 0.00 | 30.00 | 3.09 | 10.01 |
| Dohsa-hou | Block 1 | AX | 1.90 | 0.07 | 0.00 | 5.71 | 0.81 | -1.46 |
|  |  | AY | 1.67 | 0.33 | 0.00 | 20.00 | 3.46 | 12.00 |
|  |  | BX | 0.00 | 0.00 | 0.00 | 0.00 | - | - |
|  |  | BY | 0.00 | 0.00 | 0.00 | 0.00 | - | - |
|  | Block 2 | AX | 0.83 | 0.01 | 0.00 | 2.86 | 0.74 | -0.19 |
|  |  | AY | 5.83 | 0.81 | 0.00 | 30.00 | 1.95 | 4.37 |
|  |  | BX | 2.50 | 0.20 | 0.00 | 10.00 | 1.33 | -0.33 |
|  |  | BY | 1.67 | 0.15 | 0.00 | 10.00 | 2.06 | 2.64 |
|  | Block 1-2 | AX | 1.37 | 0.03 | 0.00 | 4.29 | 0.85 | -0.92 |
|  | (Pre-test) | AY | 3.75 | 0.32 | 0.00 | 15.00 | 1.47 | 0.99 |
|  |  | BX | 1.25 | 0.05 | 0.00 | 5.00 | 1.33 | -0.33 |
|  |  | BY | 0.83 | 0.04 | 0.00 | 5.00 | 2.06 | 2.64 |
|  | Block 3 | AX | 1.19 | 0.03 | 0.00 | 5.71 | 1.92 | 4.15 |
|  |  | AY | 4.17 | 0.27 | 0.00 | 10.00 | 0.39 | -2.26 |
|  |  | BX | 5.83 | 0.99 | 0.00 | 30.00 | 1.71 | 2.23 |
|  |  | BY | 1.67 | 0.15 | 0.00 | 10.00 | 2.06 | 2.64 |
|  | Block 4 | AX | 0.95 | 0.02 | 0.00 | 4.29 | 1.33 | 0.43 |
|  |  | AY | 5.83 | 0.63 | 0.00 | 20.00 | 0.99 | -0.46 |
|  |  | BX | 1.67 | 0.15 | 0.00 | 10.00 | 2.06 | 2.64 |
|  |  | BY | 0.00 | 0.00 | 0.00 | 0.00 | - | - |
|  | Block 3-4 | AX | 1.07 | 0.02 | 0.00 | 4.29 | 1.41 | 1.41 |
|  | (Post-test) | AY | 5.00 | 0.32 | 0.00 | 15.00 | 0.91 | -0.34 |
|  |  | BX | 3.75 | 0.28 | 0.00 | 15.00 | 1.15 | 0.13 |
|  |  | BY | 0.83 | 0.04 | 0.00 | 5.00 | 2.06 | 2.64 |
| Active control | Block 1 | AX | 1.87 | 0.03 | 0.00 | 5.71 | 1.08 | 1.02 |
|  |  | AY | 5.38 | 0.77 | 0.00 | 30.00 | 2.05 | 4.83 |
|  |  | BX | 1.54 | 0.14 | 0.00 | 10.00 | 2.18 | 3.22 |
|  |  | BY | 0.77 | 0.08 | 0.00 | 10.00 | 3.61 | 13.00 |
|  | Block 2 | AX | 1.65 | 0.02 | 0.00 | 4.29 | 0.26 | -0.91 |
|  |  | AY | 6.92 | 0.73 | 0.00 | 20.00 | 0.71 | -1.24 |
|  |  | BX | 3.85 | 0.76 | 0.00 | 30.00 | 2.66 | 7.47 |
|  |  | BY | 0.77 | 0.08 | 0.00 | 10.00 | 3.61 | 13.00 |
|  | Block 1-2 | AX | 1.76 | 0.02 | 0.00 | 4.29 | 0.30 | -0.82 |
|  | (Pre-test) | AY | 6.15 | 0.42 | 0.00 | 15.00 | 0.30 | -1.80 |
|  |  | BX | 2.69 | 0.19 | 0.00 | 15.00 | 2.05 | 4.83 |
|  |  | BY | 0.77 | 0.04 | 0.00 | 5.00 | 2.18 | 3.22 |
|  | Block 3 | AX | 1.54 | 0.03 | 0.00 | 4.29 | 0.68 | -0.76 |
|  |  | AY | 3.85 | 0.42 | 0.00 | 20.00 | 1.58 | 1.80 |
|  |  | BX | 3.85 | 0.42 | 0.00 | 20.00 | 1.58 | 1.80 |
|  |  | BY | 4.62 | 0.94 | 0.00 | 30.00 | 2.09 | 3.58 |
|  | Block 4 | AX | 2.31 | 0.05 | 0.00 | 7.14 | 1.31 | 1.00 |
|  |  | AY | 7.69 | 1.19 | 0.00 | 30.00 | 1.44 | 1.12 |
|  |  | BX | 3.85 | 0.42 | 0.00 | 20.00 | 1.58 | 1.80 |
|  |  | BY | 3.08 | 0.73 | 0.00 | 30.00 | 3.08 | 9.72 |
|  | Block 3-4 | AX | 1.92 | 0.03 | 0.00 | 5.71 | 1.17 | 0.30 |
|  | (Post-test) | AY | 5.77 | 0.62 | 0.00 | 25.00 | 1.83 | 2.68 |
|  |  | BX | 3.85 | 0.34 | 0.00 | 15.00 | 1.27 | 0.15 |
|  |  | BY | 3.85 | 0.80 | 0.00 | 30.00 | 2.59 | 6.58 |
| Passive control | Block 1 | AX | 2.75 | 0.18 | 0.00 | 17.14 | 2.21 | 5.09 |
|  |  | AY | 3.46 | 0.48 | 0.00 | 30.00 | 2.59 | 8.08 |
|  |  | BX | 3.46 | 0.56 | 0.00 | 30.00 | 2.46 | 6.19 |
|  |  | BY | 2.69 | 0.44 | 0.00 | 30.00 | 3.15 | 11.24 |
|  | Block 2 | AX | 2.97 | 0.23 | 0.00 | 20.00 | 2.38 | 6.03 |
|  |  | AY | 8.85 | 1.79 | 0.00 | 50.00 | 1.86 | 3.18 |
|  |  | BX | 5.00 | 0.90 | 0.00 | 40.00 | 2.44 | 6.75 |
|  |  | BY | 1.92 | 0.48 | 0.00 | 30.00 | 3.61 | 12.59 |
|  | Block 1-2 | AX | 2.86 | 0.20 | 0.00 | 18.57 | 2.46 | 6.30 |
|  | (Pre-test) | AY | 6.15 | 0.77 | 0.00 | 30.00 | 1.60 | 1.81 |
|  |  | BX | 4.23 | 0.49 | 0.00 | 25.00 | 1.79 | 2.47 |
|  |  | BY | 2.31 | 0.26 | 0.00 | 20.00 | 2.50 | 5.91 |
|  | Block 3 | AX | 2.64 | 0.06 | 0.00 | 11.43 | 1.88 | 4.87 |
|  |  | AY | 10.38 | 1.72 | 0.00 | 50.00 | 1.54 | 2.50 |
|  |  | BX | 4.23 | 0.65 | 0.00 | 30.00 | 1.99 | 3.45 |
|  |  | BY | 2.69 | 0.68 | 0.00 | 40.00 | 4.03 | 17.74 |
|  | Block 4 | AX | 2.91 | 0.14 | 0.00 | 15.71 | 1.93 | 4.59 |
|  |  | AY | 14.23 | 1.77 | 0.00 | 40.00 | 0.67 | -0.51 |
|  |  | BX | 8.08 | 0.96 | 0.00 | 40.00 | 1.80 | 3.96 |
|  |  | BY | 3.08 | 0.46 | 0.00 | 30.00 | 2.85 | 9.47 |
|  | Block 3-4 | AX | 2.77 | 0.07 | 0.00 | 10.71 | 1.59 | 2.54 |
|  | (Post-test) | AY | 12.31 | 1.22 | 0.00 | 45.00 | 1.16 | 1.44 |
|  |  | BX | 6.15 | 0.55 | 0.00 | 25.00 | 1.42 | 1.47 |
|  |  | BY | 2.88 | 0.34 | 0.00 | 25.00 | 2.71 | 8.02 |

**Supplementary** **Table 2.** Descriptive statistics for each group on the response times of the AX-CPT.

| Group | Block | Trail type | Mean RT | Variance | Min | Max | Skew | Kurtosis |
| --- | --- | --- | --- | --- | --- | --- | --- | --- |
| All | Block 1 | AX | 510.20 | 27.32 | 311.53 | 928.87 | 0.79 | -0.49 |
|  |  | AY | 584.76 | 20.30 | 400.66 | 860.74 | 0.46 | -1.17 |
|  |  | BX | 480.28 | 34.40 | 250.84 | 940.79 | 0.69 | -0.53 |
|  |  | BY | 465.74 | 33.98 | 247.44 | 958.68 | 0.79 | -0.51 |
|  | Block 2 | AX | 505.16 | 29.94 | 302.07 | 968.50 | 1.04 | 0.30 |
|  |  | AY | 592.16 | 23.05 | 413.33 | 1008.18 | 0.93 | 0.15 |
|  |  | BX | 454.95 | 36.94 | 216.47 | 893.34 | 0.76 | -0.86 |
|  |  | BY | 466.03 | 37.72 | 231.67 | 916.43 | 0.72 | -0.84 |
|  | Block 1-2 | AX | 507.64 | 27.36 | 312.93 | 947.53 | 0.93 | 0.03 |
|  | (Pre-test) | AY | 588.34 | 19.97 | 417.13 | 926.81 | 0.71 | -0.47 |
|  |  | BX | 467.99 | 34.08 | 237.46 | 917.06 | 0.74 | -0.71 |
|  |  | BY | 466.39 | 33.51 | 239.97 | 898.50 | 0.75 | -0.70 |
|  | Block 3 | AX | 441.21 | 19.94 | 304.33 | 829.98 | 1.43 | 0.99 |
|  |  | AY | 540.78 | 14.49 | 400.02 | 884.11 | 1.26 | 0.83 |
|  |  | BX | 385.69 | 26.47 | 158.59 | 796.19 | 1.22 | 0.62 |
|  |  | BY | 397.31 | 24.54 | 192.54 | 825.18 | 1.08 | 0.60 |
|  | Block 4 | AX | 450.33 | 19.68 | 301.71 | 968.86 | 1.80 | 3.42 |
|  |  | AY | 554.19 | 15.62 | 414.90 | 954.32 | 1.45 | 1.72 |
|  |  | BX | 382.28 | 33.17 | 216.30 | 966.30 | 1.76 | 2.58 |
|  |  | BY | 379.75 | 30.57 | 201.80 | 938.24 | 1.58 | 1.79 |
|  | Block 3-4 | AX | 445.64 | 19.05 | 303.01 | 899.42 | 1.59 | 2.02 |
|  | (Post-test) | AY | 547.77 | 14.44 | 414.45 | 896.20 | 1.36 | 1.16 |
|  |  | BX | 384.69 | 28.50 | 203.78 | 881.25 | 1.52 | 1.62 |
|  |  | BY | 388.52 | 25.70 | 214.87 | 835.13 | 1.33 | 0.91 |
| Dohsa-hou | Block 1 | AX | 479.03 | 23.37 | 311.53 | 725.70 | 0.66 | -1.49 |
|  |  | AY | 543.63 | 16.95 | 421.20 | 760.17 | 0.78 | -1.37 |
|  |  | BX | 463.20 | 31.55 | 258.46 | 708.67 | 0.30 | -1.74 |
|  |  | BY | 439.36 | 32.20 | 247.44 | 696.07 | 0.48 | -1.58 |
|  | Block 2 | AX | 467.80 | 18.57 | 314.27 | 727.04 | 0.63 | -0.91 |
|  |  | AY | 552.57 | 12.29 | 435.04 | 716.51 | 0.53 | -1.48 |
|  |  | BX | 427.62 | 28.93 | 216.47 | 716.44 | 0.40 | -1.18 |
|  |  | BY | 443.94 | 30.14 | 231.67 | 750.86 | 0.42 | -1.20 |
|  | Block 1-2 | AX | 473.37 | 20.47 | 312.93 | 726.39 | 0.66 | -1.23 |
|  | (Pre-test) | AY | 547.92 | 14.31 | 431.87 | 732.94 | 0.69 | -1.45 |
|  |  | BX | 445.53 | 29.39 | 237.46 | 712.55 | 0.39 | -1.53 |
|  |  | BY | 441.54 | 30.00 | 239.97 | 716.31 | 0.40 | -1.52 |
|  | Block 3 | AX | 404.57 | 14.70 | 315.24 | 702.45 | 1.76 | 2.51 |
|  |  | AY | 492.21 | 8.72 | 400.02 | 703.98 | 1.13 | 0.79 |
|  |  | BX | 366.65 | 21.38 | 233.71 | 725.79 | 1.72 | 2.51 |
|  |  | BY | 371.39 | 30.21 | 203.99 | 766.46 | 1.20 | 0.91 |
|  | Block 4 | AX | 423.04 | 12.62 | 327.88 | 689.27 | 1.55 | 1.82 |
|  |  | AY | 515.07 | 6.24 | 427.44 | 669.83 | 0.65 | -0.49 |
|  |  | BX | 360.66 | 23.25 | 221.14 | 686.89 | 1.16 | 0.23 |
|  |  | BY | 345.73 | 22.52 | 225.75 | 657.10 | 1.30 | 0.15 |
|  | Block 3-4 | AX | 413.82 | 13.52 | 321.56 | 695.81 | 1.68 | 2.25 |
|  | (Post-test) | AY | 503.76 | 7.18 | 414.45 | 686.90 | 0.94 | 0.32 |
|  |  | BX | 363.60 | 21.91 | 228.22 | 706.34 | 1.46 | 1.35 |
|  |  | BY | 358.50 | 25.65 | 214.87 | 711.78 | 1.26 | 0.58 |
| Active Control | Block 1 | AX | 509.85 | 33.03 | 330.32 | 873.96 | 1.01 | -0.10 |
|  |  | AY | 590.69 | 25.37 | 416.50 | 856.95 | 0.64 | -1.14 |
|  |  | BX | 473.08 | 41.21 | 250.84 | 866.19 | 0.79 | -0.40 |
|  |  | BY | 462.39 | 47.22 | 263.21 | 958.68 | 1.30 | 0.85 |
|  | Block 2 | AX | 539.43 | 44.04 | 323.51 | 968.50 | 0.82 | -0.48 |
|  |  | AY | 607.36 | 31.91 | 413.33 | 986.85 | 0.90 | -0.06 |
|  |  | BX | 473.60 | 43.84 | 257.05 | 780.67 | 0.59 | -1.52 |
|  |  | BY | 502.51 | 47.48 | 256.93 | 838.31 | 0.35 | -1.62 |
|  | Block 1-2 | AX | 524.55 | 37.28 | 329.39 | 921.23 | 0.93 | -0.21 |
|  | (Pre-test) | AY | 599.34 | 25.58 | 427.22 | 921.90 | 0.82 | -0.17 |
|  |  | BX | 473.36 | 40.80 | 266.38 | 822.49 | 0.69 | -1.05 |
|  |  | BY | 482.55 | 44.29 | 271.10 | 898.50 | 0.85 | -0.56 |
|  | Block 3 | AX | 477.66 | 30.27 | 308.82 | 829.98 | 1.03 | -0.16 |
|  |  | AY | 557.09 | 16.72 | 432.26 | 838.08 | 1.38 | 1.04 |
|  |  | BX | 395.63 | 41.34 | 158.59 | 796.19 | 1.06 | 0.26 |
|  |  | BY | 417.53 | 30.51 | 192.54 | 759.54 | 0.92 | 0.19 |
|  | Block 4 | AX | 477.86 | 38.65 | 315.67 | 968.86 | 1.77 | 2.52 |
|  |  | AY | 579.89 | 25.07 | 442.60 | 954.32 | 1.77 | 2.35 |
|  |  | BX | 421.24 | 57.22 | 229.90 | 966.30 | 1.74 | 1.91 |
|  |  | BY | 419.57 | 46.46 | 241.47 | 938.24 | 1.57 | 1.67 |
|  | Block 3-4 | AX | 477.78 | 33.37 | 312.22 | 899.42 | 1.43 | 1.27 |
|  | (Post-test) | AY | 568.47 | 20.06 | 445.01 | 896.20 | 1.70 | 2.08 |
|  |  | BX | 408.40 | 48.23 | 203.78 | 881.25 | 1.48 | 1.16 |
|  |  | BY | 418.56 | 36.30 | 230.62 | 835.13 | 1.31 | 0.83 |
| Passive control | Block 1 | AX | 524.76 | 27.82 | 327.16 | 928.87 | 0.77 | -0.38 |
|  |  | AY | 600.77 | 19.87 | 400.66 | 860.74 | 0.26 | -1.17 |
|  |  | BX | 491.77 | 34.84 | 259.55 | 940.79 | 0.85 | -0.14 |
|  |  | BY | 479.59 | 30.58 | 270.41 | 803.67 | 0.64 | -1.13 |
|  | Block 2 | AX | 505.27 | 29.30 | 302.07 | 966.75 | 1.18 | 0.71 |
|  |  | AY | 602.82 | 24.39 | 425.58 | 1008.18 | 0.88 | 0.03 |
|  |  | BX | 458.23 | 39.55 | 265.03 | 893.34 | 0.95 | -0.62 |
|  |  | BY | 458.00 | 38.39 | 259.19 | 916.43 | 1.02 | -0.28 |
|  | Block 1-2 | AX | 515.01 | 27.05 | 314.61 | 947.53 | 1.00 | 0.38 |
|  | (Pre-test) | AY | 601.50 | 20.35 | 417.13 | 926.81 | 0.60 | -0.60 |
|  |  | BX | 475.68 | 35.33 | 264.10 | 917.06 | 0.91 | -0.39 |
|  |  | BY | 469.78 | 32.12 | 280.27 | 860.05 | 0.84 | -0.70 |
|  | Block 3 | AX | 439.90 | 17.54 | 304.33 | 765.33 | 1.66 | 1.90 |
|  |  | AY | 555.03 | 15.63 | 414.75 | 884.11 | 1.22 | 0.63 |
|  |  | BX | 389.50 | 23.45 | 237.83 | 784.50 | 1.25 | 0.78 |
|  |  | BY | 399.17 | 20.59 | 230.93 | 825.18 | 1.35 | 1.85 |
|  | Block 4 | AX | 449.17 | 14.49 | 301.71 | 814.19 | 1.39 | 2.01 |
|  |  | AY | 559.40 | 15.37 | 414.90 | 810.85 | 0.93 | -0.38 |
|  |  | BX | 372.78 | 27.53 | 216.30 | 876.50 | 1.78 | 2.87 |
|  |  | BY | 375.54 | 27.53 | 201.80 | 848.06 | 1.63 | 2.03 |
|  | Block 3-4 | AX | 444.25 | 15.10 | 303.01 | 787.60 | 1.50 | 1.76 |
|  | (Post-test) | AY | 557.74 | 14.83 | 430.84 | 845.14 | 1.05 | -0.04 |
|  |  | BX | 382.56 | 23.71 | 236.67 | 828.08 | 1.49 | 1.83 |
|  |  | BY | 387.35 | 21.78 | 221.55 | 766.61 | 1.51 | 1.58 |

**Supplementary** **Table 3**. Alpha coefficients and ICCs of the AX-CPT.

| Group | Trial Type | B1 | B2 | Pre | B1-2 | B3 | B4 | Post | B3-4 |
| --- | --- | --- | --- | --- | --- | --- | --- | --- | --- |
|  |  | *α* | *α* | *α* | *ICC* | *α* | *α* | *α* | *ICC* |
| All | AX | .73 | .79 | .87 | .92 | .42 | .67 | .71 | .62 |
|  | AY | .37 | .50 | .57 | .41 | .44 | .39 | .61 | .63 |
|  | BX | .46 | .48 | .57 | .40 | .37 | .27 | .47 | .44 |
|  | BY | .45 | .57 | .54 | .24 | .61 | .52 | .71 | .65 |
| Dohsa-hou | AX | .65 | -.36 | .50 | .57 | .46 | .48 | .63 | .64 |
|  | AY | 1.00 | .36 | .22 | .15 | -.69 | .12 | .26 | .61 |
|  | BX | - | -.33 | .00 | - | .55 | -.20 | .41 | .00 |
|  | BY | - | - | .00 | - | -.20 | - | -.20 | .00 |
| Active control | AX | .10 | -.18 | .64 | .47 | .14 | .32 | .58 | .82 |
|  | AY | .38 | .15 | .27 | .13 | .16 | .48 | .62 | .67 |
|  | BX | -.18 | .68 | .00 | -.20 | .11 | .11 | .48 | .77 |
|  | BY | - | - | .00 | -.08 | .61 | .77 | .84 | .95 |
| Passive control | AX | .81 | .85 | .95 | .92 | .43 | .73 | .72 | .53 |
|  | AY | .34 | .61 | .49 | .44 | .51 | .34 | .59 | .57 |
|  | BX | .25 | .57 | .53 | .37 | .46 | .24 | .50 | .50 |
|  | BY | .48 | .75 | .27 | .14 | .73 | .42 | .64 | .36 |
| *Note.* Pre Test ＝ Block 1 & 2, Post Test = Block 3 & 4. | | | | | | |  |  |  |

**Supplementary** **Table 4.** Descriptive statistics for each group on the error rates of the Modified Stroop Task.

| Group | Block | Trail type | Mean ER | Variance | Min | Max | Skew | Kurtosis |
| --- | --- | --- | --- | --- | --- | --- | --- | --- |
| All | Block 1 | Congruent | 4.28 | 0.26 | 0.00 | 25.00 | 2.42 | 6.45 |
|  |  | Incongruent | 10.53 | 0.89 | 0.00 | 45.83 | 1.40 | 2.44 |
|  | Block 2 | Congruent | 3.98 | 0.23 | 0.00 | 23.21 | 2.22 | 5.56 |
|  |  | Incongruent | 11.79 | 0.85 | 0.00 | 37.50 | 0.72 | 0.05 |
|  | Block 3 | Congruent | 6.00 | 0.69 | 0.00 | 33.93 | 2.18 | 4.44 |
|  |  | Incongruent | 11.87 | 0.95 | 0.00 | 37.50 | 0.80 | 0.15 |
|  | Block 1-3 | Congruent | 4.75 | 0.30 | 0.60 | 26.79 | 2.36 | 5.63 |
|  | (Pre-test) | Incongruent | 11.40 | 0.58 | 0.00 | 27.78 | 0.50 | -0.97 |
|  | Block 4 | Congruent | 4.01 | 0.31 | 0.00 | 35.71 | 3.93 | 20.56 |
|  |  | Incongruent | 8.88 | 0.53 | 0.00 | 29.17 | 0.99 | 0.67 |
|  | Block 5 | Congruent | 4.75 | 0.38 | 0.00 | 37.50 | 3.27 | 14.66 |
|  |  | Incongruent | 9.12 | 0.59 | 0.00 | 33.33 | 1.14 | 1.86 |
|  | Block6 | Congruent | 4.48 | 0.18 | 0.00 | 21.43 | 1.70 | 4.32 |
|  |  | Incongruent | 9.98 | 0.82 | 0.00 | 45.83 | 1.34 | 3.28 |
|  | Block 4-6 | Congruent | 4.41 | 0.24 | 0.00 | 31.55 | 3.53 | 17.18 |
|  | (Post-test) | Incongruent | 9.33 | 0.45 | 0.00 | 36.11 | 1.26 | 3.44 |
| Dohsa-hou | Block 1 | Congruent | 3.02 | 0.13 | 0.00 | 14.29 | 2.88 | 9.44 |
|  |  | Incongruent | 8.65 | 0.79 | 0.00 | 25.00 | 0.85 | -0.91 |
|  | Block 2 | Congruent | 3.71 | 0.14 | 0.00 | 12.50 | 1.35 | 1.18 |
|  |  | Incongruent | 11.54 | 1.10 | 0.00 | 33.33 | 0.82 | -0.30 |
|  | Block 3 | Congruent | 3.85 | 0.22 | 0.00 | 17.86 | 2.44 | 7.05 |
|  |  | Incongruent | 9.62 | 0.62 | 0.00 | 20.83 | 0.18 | -1.53 |
|  | Block 1-3 | Congruent | 3.53 | 0.14 | 0.60 | 14.88 | 2.60 | 7.52 |
|  | (Pre-test) | Incongruent | 9.94 | 0.71 | 1.39 | 25.00 | 0.80 | -1.18 |
|  | Block 4 | Congruent | 3.16 | 0.19 | 0.00 | 16.07 | 2.43 | 6.70 |
|  |  | Incongruent | 8.33 | 0.46 | 0.00 | 20.83 | 0.41 | -0.75 |
|  | Block 5 | Congruent | 2.75 | 0.12 | 0.00 | 12.50 | 1.99 | 5.03 |
|  |  | Incongruent | 6.41 | 0.34 | 0.00 | 16.67 | 0.78 | -0.42 |
|  | Block6 | Congruent | 3.71 | 0.06 | 0.00 | 7.14 | -0.16 | -0.78 |
|  |  | Incongruent | 6.73 | 0.33 | 0.00 | 16.67 | 0.16 | -1.28 |
|  | Block 4-6 | Congruent | 3.21 | 0.09 | 0.00 | 11.31 | 1.84 | 4.74 |
|  | (Post-test) | Incongruent | 7.16 | 0.25 | 1.39 | 16.67 | 0.59 | -0.79 |
| Active control | Block 1 | Congruent | 3.42 | 0.19 | 0.00 | 14.29 | 1.73 | 2.45 |
|  |  | Incongruent | 13.89 | 2.00 | 0.00 | 45.83 | 1.28 | 0.81 |
|  | Block 2 | Congruent | 3.72 | 0.21 | 0.00 | 16.07 | 2.13 | 4.92 |
|  |  | Incongruent | 12.15 | 0.99 | 0.00 | 29.17 | 0.21 | -1.04 |
|  | Block 3 | Congruent | 4.76 | 0.48 | 0.00 | 23.21 | 2.01 | 4.12 |
|  |  | Incongruent | 13.19 | 0.85 | 4.17 | 37.50 | 1.81 | 4.05 |
|  | Block 1-3 | Congruent | 3.97 | 0.23 | 0.60 | 16.07 | 1.90 | 3.21 |
|  | (Pre-test) | Incongruent | 13.08 | 0.60 | 4.17 | 23.61 | 0.28 | -1.46 |
|  | Block 4 | Congruent | 3.87 | 0.16 | 0.00 | 12.50 | 1.02 | 0.25 |
|  |  | Incongruent | 11.46 | 0.76 | 0.00 | 29.17 | 0.61 | 0.08 |
|  | Block 5 | Congruent | 5.36 | 0.24 | 1.79 | 17.86 | 1.83 | 3.24 |
|  |  | Incongruent | 11.46 | 0.45 | 0.00 | 20.83 | -0.47 | -1.15 |
|  | Block6 | Congruent | 3.13 | 0.14 | 0.00 | 12.50 | 1.61 | 2.73 |
|  |  | Incongruent | 11.81 | 0.63 | 0.00 | 25.00 | 0.29 | 0.01 |
|  | Block 4-6 | Congruent | 4.12 | 0.17 | 0.60 | 14.29 | 1.64 | 2.53 |
|  | (Post-test) | Incongruent | 11.57 | 0.44 | 0.00 | 20.83 | -0.35 | -1.08 |
| Passive control | Block 1 | Congruent | 5.23 | 0.35 | 0.00 | 25.00 | 2.33 | 5.55 |
|  |  | Incongruent | 9.97 | 0.48 | 0.00 | 25.00 | 0.57 | -0.45 |
|  | Block 2 | Congruent | 4.21 | 0.29 | 0.00 | 23.21 | 2.36 | 6.07 |
|  |  | Incongruent | 11.76 | 0.75 | 0.00 | 37.50 | 1.04 | 1.52 |
|  | Block 3 | Congruent | 7.53 | 0.98 | 0.00 | 33.93 | 1.86 | 2.63 |
|  |  | Incongruent | 12.35 | 1.18 | 0.00 | 33.33 | 0.63 | -0.62 |
|  | Block 1-3 | Congruent | 5.65 | 0.41 | 0.60 | 26.79 | 2.22 | 4.55 |
|  | (Pre-test) | Incongruent | 11.36 | 0.53 | 0.00 | 27.78 | 0.57 | -0.36 |
|  | Block 4 | Congruent | 4.46 | 0.44 | 0.00 | 35.71 | 4.16 | 19.94 |
|  |  | Incongruent | 8.04 | 0.46 | 0.00 | 29.17 | 1.45 | 2.25 |
|  | Block 5 | Congruent | 5.42 | 0.56 | 0.00 | 37.50 | 3.17 | 12.50 |
|  |  | Incongruent | 9.38 | 0.74 | 0.00 | 33.33 | 1.48 | 2.63 |
|  | Block6 | Congruent | 5.42 | 0.24 | 0.00 | 21.43 | 1.54 | 3.17 |
|  |  | Incongruent | 10.71 | 1.11 | 0.00 | 45.83 | 1.44 | 3.12 |
|  | Block 4-6 | Congruent | 5.10 | 0.35 | 0.60 | 31.55 | 3.52 | 15.17 |
|  | (Post-test) | Incongruent | 9.37 | 0.53 | 0.00 | 36.11 | 1.82 | 5.80 |

**Supplementary** **Table 5.** Descriptive statistics for each group on the response times of the Modified Stroop Task.

| Group | Block | Trail type | Mean ER | Variance | Min | Max | Skew | Kurtosis |
| --- | --- | --- | --- | --- | --- | --- | --- | --- |
| All | Block 1 | Congruent | 564.13 | 4.18 | 410.52 | 741.43 | 0.37 | 1.16 |
|  |  | Incongruent | 623.94 | 5.68 | 430.67 | 786.69 | -0.02 | 0.06 |
|  | Block 2 | Congruent | 552.77 | 3.17 | 397.08 | 684.75 | -0.20 | 0.88 |
|  |  | Incongruent | 621.75 | 4.38 | 406.03 | 755.85 | -0.46 | 1.11 |
|  | Block 3 | Congruent | 552.88 | 4.30 | 398.14 | 739.54 | 0.34 | 0.90 |
|  |  | Incongruent | 618.40 | 6.16 | 427.42 | 798.17 | -0.01 | 0.08 |
|  | Block 1-3 | Congruent | 556.47 | 3.43 | 402.00 | 715.64 | 0.14 | 1.20 |
|  | (Pre-test) | Incongruent | 620.99 | 4.26 | 448.29 | 749.90 | -0.28 | 0.37 |
|  | Block 4 | Congruent | 539.28 | 3.61 | 391.89 | 659.10 | -0.19 | 0.03 |
|  |  | Incongruent | 596.55 | 4.24 | 434.35 | 727.74 | -0.17 | 0.16 |
|  | Block 5 | Congruent | 546.18 | 4.26 | 406.91 | 715.33 | 0.54 | -0.01 |
|  |  | Incongruent | 598.87 | 5.49 | 433.34 | 758.17 | 0.21 | -0.24 |
|  | Block6 | Congruent | 551.31 | 4.91 | 406.20 | 752.91 | 0.57 | 0.37 |
|  |  | Incongruent | 602.04 | 5.36 | 460.01 | 783.65 | 0.45 | -0.26 |
|  | Block 4-6 | Congruent | 545.55 | 3.86 | 401.94 | 692.12 | 0.26 | -0.08 |
|  | (Post-test) | Incongruent | 599.02 | 4.23 | 449.13 | 737.74 | 0.10 | 0.01 |
| Dohsa-hou | Block 1 | Congruent | 569.97 | 3.90 | 434.56 | 677.39 | -0.26 | 0.99 |
|  |  | Incongruent | 600.90 | 4.22 | 430.67 | 697.21 | -1.21 | 3.62 |
|  | Block 2 | Congruent | 551.46 | 3.21 | 410.72 | 637.87 | -1.11 | 2.39 |
|  |  | Incongruent | 615.05 | 4.66 | 486.96 | 755.85 | 0.27 | 0.80 |
|  | Block 3 | Congruent | 555.25 | 3.93 | 411.00 | 624.76 | -0.91 | 0.79 |
|  |  | Incongruent | 610.26 | 6.69 | 427.42 | 733.67 | -0.71 | 0.81 |
|  | Block 1-3 | Congruent | 558.85 | 3.34 | 418.81 | 643.94 | -0.93 | 1.84 |
|  | (Pre-test) | Incongruent | 608.38 | 4.16 | 448.29 | 689.60 | -1.10 | 2.11 |
|  | Block 4 | Congruent | 540.26 | 3.73 | 391.97 | 612.61 | -1.23 | 1.60 |
|  |  | Incongruent | 590.94 | 4.42 | 434.35 | 713.04 | -0.71 | 2.11 |
|  | Block 5 | Congruent | 550.70 | 2.92 | 439.38 | 624.47 | -0.38 | -0.25 |
|  |  | Incongruent | 590.55 | 6.60 | 451.73 | 724.72 | 0.07 | -0.45 |
|  | Block6 | Congruent | 559.91 | 3.33 | 434.33 | 642.71 | -0.73 | 0.59 |
|  |  | Incongruent | 610.07 | 6.36 | 460.01 | 783.65 | 0.32 | 1.17 |
|  | Block 4-6 | Congruent | 550.26 | 3.07 | 422.63 | 626.41 | -0.94 | 0.98 |
|  | (Post-test) | Incongruent | 597.33 | 4.92 | 449.13 | 737.74 | -0.14 | 1.42 |
| Active control | Block 1 | Congruent | 543.16 | 2.06 | 445.96 | 591.13 | -1.14 | 0.38 |
|  |  | Incongruent | 611.57 | 5.17 | 505.50 | 746.27 | 0.17 | -0.18 |
|  | Block 2 | Congruent | 548.65 | 1.87 | 475.20 | 622.33 | -0.08 | -0.29 |
|  |  | Incongruent | 616.39 | 2.11 | 524.32 | 689.95 | -0.54 | 0.05 |
|  | Block 3 | Congruent | 542.22 | 2.16 | 456.42 | 619.05 | -0.05 | -0.48 |
|  |  | Incongruent | 605.85 | 1.79 | 536.96 | 681.18 | 0.39 | -0.13 |
|  | Block 1-3 | Congruent | 544.56 | 1.60 | 474.22 | 602.66 | -0.58 | -0.77 |
|  | (Pre-test) | Incongruent | 609.58 | 1.75 | 533.58 | 668.03 | -0.30 | -0.56 |
|  | Block 4 | Congruent | 526.08 | 3.52 | 444.95 | 659.10 | 0.92 | 1.04 |
|  |  | Incongruent | 590.25 | 2.93 | 477.63 | 664.62 | -0.78 | 0.10 |
|  | Block 5 | Congruent | 541.88 | 5.90 | 462.70 | 715.33 | 1.12 | 0.82 |
|  |  | Incongruent | 606.55 | 6.10 | 510.40 | 754.10 | 0.79 | -0.33 |
|  | Block6 | Congruent | 542.61 | 4.65 | 456.31 | 694.60 | 1.01 | 1.10 |
|  |  | Incongruent | 604.76 | 4.40 | 512.01 | 763.40 | 1.09 | 2.13 |
|  | Block 4-6 | Congruent | 536.91 | 4.18 | 458.87 | 660.04 | 0.82 | -0.30 |
|  | (Post-test) | Incongruent | 600.32 | 3.73 | 500.51 | 719.16 | 0.34 | -0.10 |
| Passive control | Block 1 | Congruent | 570.41 | 5.22 | 410.52 | 741.43 | 0.50 | 0.97 |
|  |  | Incongruent | 639.93 | 6.37 | 491.86 | 786.69 | -0.01 | -0.57 |
|  | Block 2 | Congruent | 555.15 | 3.91 | 397.08 | 684.75 | 0.00 | 0.64 |
|  |  | Incongruent | 627.16 | 5.45 | 406.03 | 732.83 | -0.80 | 1.47 |
|  | Block 3 | Congruent | 556.35 | 5.59 | 398.14 | 739.54 | 0.57 | 0.75 |
|  |  | Incongruent | 627.57 | 7.98 | 460.25 | 798.17 | 0.01 | -0.57 |
|  | Block 1-3 | Congruent | 560.47 | 4.39 | 402.00 | 715.64 | 0.35 | 0.96 |
|  | (Pre-test) | Incongruent | 631.73 | 5.40 | 456.16 | 749.90 | -0.28 | -0.25 |
|  | Block 4 | Congruent | 544.47 | 3.75 | 391.89 | 650.09 | -0.20 | 0.10 |
|  |  | Incongruent | 601.85 | 4.94 | 447.94 | 727.74 | 0.03 | -0.38 |
|  | Block 5 | Congruent | 545.94 | 4.47 | 406.91 | 678.12 | 0.52 | -0.03 |
|  |  | Incongruent | 599.43 | 5.10 | 433.34 | 758.17 | 0.06 | 0.18 |
|  | Block6 | Congruent | 551.05 | 6.01 | 406.20 | 752.91 | 0.75 | 0.50 |
|  |  | Incongruent | 597.15 | 5.65 | 476.78 | 730.42 | 0.40 | -1.06 |
|  | Block 4-6 | Congruent | 547.07 | 4.32 | 401.94 | 692.12 | 0.41 | 0.23 |
|  | (Post-test) | Incongruent | 599.24 | 4.43 | 451.01 | 736.39 | 0.16 | -0.19 |

**Supplementary** **Table 6.** Alpha coefficients and ICCs of the Modified Stroop Task.

| Group | Trial Type | B1 | B2 | B3 | Pre | B1-3 | | B4 | B5 | B6 | Post | B4-6 |
| --- | --- | --- | --- | --- | --- | --- | --- | --- | --- | --- | --- | --- |
|  |  | *α* | *α* | *α* | *α* | *ICC* | *α* | | *α* | *α* | *α* | *ICC* |
| All | Congruent | .73 | .71 | .87 | .92 | .85 | .79 | | .80 | .59 | .90 | .91 |
|  | Incongruent | .58 | .52 | .56 | .77 | .72 | .38 | | .43 | .57 | .75 | .78 |
| Dohsa-hou | Congruent | .63 | .56 | .73 | .87 | .93 | .75 | | .64 | -.14 | .80 | .79 |
|  | Incongruent | .63 | .66 | .42 | .84 | .91 | .34 | | .26 | .22 | .64 | .75 |
| Active control | Congruent | .72 | .73 | .87 | .92 | .86 | .61 | | .64 | .64 | .87 | .94 |
|  | Incongruent | .78 | .59 | .46 | .75 | .47 | .50 | | .02 | .31 | .69 | .82 |
| Passive control | Congruent | .76 | .77 | .89 | .93 | .83 | .85 | | .85 | .64 | .93 | .91 |
|  | Incongruent | .22 | .44 | .65 | .75 | .74 | .35 | | .54 | .67 | .79 | .77 |
| *Note.* Pre-test ＝ Block 1 & 2, Post Test = Block 3 & 4. | | | | | | | | | | | |  |
